# Chemically labeled toxins or bioactive peptides show a heterogeneous intracellular distribution and low spatial overlap with autofluorescence in bloom-forming cyanobacteria

**DOI:** 10.1038/s41598-020-59381-w

**Published:** 2020-02-17

**Authors:** Rainer Kurmayer, Elisabeth Entfellner, Thomas Weisse, Martin Offterdinger, Andrea Rentmeister, Li Deng

**Affiliations:** 1 University of Innsbruck, Research Department for Limnology, Mondseestrasse 9, 5310 Mondsee, Austria; 2 Innsbruck Medical University, Division of Neurobiochemistry, Biooptics Core Facility, Innrain 80, 6020 Innsbruck, Austria; 3 University of Muenster, Department of Chemistry, Institute of Biochemistry, Wilhelm-Klemm-Strasse 2, 48149 Muenster, Germany; 4 Helmholtz Centre Munich, Institute of Virology, Ingolstaedter Landstrasse 1, 85764 Neuherberg, Germany

**Keywords:** Chemical modification, Fluorescence imaging, Water microbiology, Limnology

## Abstract

Harmful algal blooms formed by colony-forming cyanobacteria deteriorate water resources by producing cyanotoxins, which frequently occur at high intracellular concentrations. We aimed to localize toxic microcystins (MCs) and bioactive anabaenopeptins (APs) at the subcellular level under noninvasive conditions. Since both metabolites are synthesized nonribosomally, the relaxed specificity of key enzymes catalyzing substrate activation allowed chemical labeling through a standard copper-catalyzed click chemistry reaction. The genera *Planktothrix* and *Microcystis* specifically incorporated unnatural amino acids such as *N*-propargyloxy-carbonyl-L-lysine or *O*-propargyl-L-tyrosine, resulting in modified AP or MC peptides carrying the incorporated alkyne moiety. The labeled cells were quantitatively differentiated from the unlabeled control cells. MCs and APs occurred intracellularly as distinct entities showing a cell-wide distribution but a lowered spatial overlap with natural autofluorescence. Using the immunofluorescence technique, colocalization with markers of individual organelles was utilized to relate the distribution of labeled MCs to cellular compartments, e.g., using RbcL and FtsZ (cytosol) and PsbA (thylakoids). The colocalization correlation coefficients calculated pairwise between organelles and autofluorescence were highly positive as opposed to the relatively low positive indices derived from labeled MCs. The lower correlation coefficients imply that only a portion of the labeled MC molecules were related spatially to the organelles in the cell.

## Introduction

Bloom-forming cyanobacteria, such as those of the genera *Microcystis* and *Planktothrix*, frequently dominate aquatic ecosystems and have received much attention due to their ability to produce various toxic or bioactive secondary metabolites, e.g., toxic microcystins (MCs) and bioactive anabaenopeptins (APs)^1,2^. In addition to their relevance for water management, these peptide families represent bioactive compounds with potential antibacterial, fungal or cytostatic effects^3^. For both *Planktothrix* and *Microcystis*, genotypes either lacking or containing intracellular toxic/bioactive peptides coexist in a local environment. Since the elucidation of the genetic basis of toxic/bioactive peptide synthesis, it has been known that both MCs and APs are synthesized by large multifunctional enzyme complexes by the thiotemplate mechanism in a stepwise manner, i.e., nonribosomal peptide synthetase (NRPS)^4,5^. Aminoacyl adenylation (A) domains are responsible for substrate recognition and activation during peptide synthesis and show a high degree of conservation, enabling the definition of general rules for the structural basis of substrate recognition^6^. For MCs and APs, so-called promiscuous A domains showing relaxed substrate specificity have been observed; this relaxed specificity results in genotypes that produce several structural variants resulting from chemically rather distinct substrates, i.e., Leu or Arg or Tyr^7–9^. For AP synthesis, this relaxed substrate specificity was subsequently characterized both *in vitro* and by crystallographic analysis, showing that only a few critical substitutions cause a conformational change during substrate activation resulting in the observed promiscuity^10^.

In addition, strains carrying genes for MC vs AP synthesis differ in toxin/bioactive peptide production by an order of magnitude when grown under standardized conditions in the laboratory^11,12^. For the best-studied toxin, MC-LR, a solubility of 1 gram per liter water (equal to 1 mM) has been estimated^13^, although increased turbidity in water caused by colloidal solution above 8 mg (8 µM) has been observed^14^. Notably, relatively high concentrations of MC and AP peptides, e.g., in the millimolar range, that partly exceed the solubility threshold of 1 mM have been reported in cells according to *in vitro* studies (e.g., Fig. 3 in^15^, Fig. 2 in^16^, and Table 1 in^12^). Although increasing evidence supports compartmentation or chemical binding of MC-LR to other (intercellular) proteins^14,17^, the challenges associated with determining the localization of MCs and other bioactive peptides in a noninvasive manner limit our ability to understand the mechanism that enables such high intracellular concentrations. Understanding this mechanism is essential for water management and risk assessment of potentially toxic cyanobacterial blooms.

In particular, the subcellular compartment in which NRPS, a large multifunctional enzyme complex for toxin synthesis, is located and which compartment is used for storage of synthesized peptides in the cells are still unclear. Cyanobacterial cells are composed of three different subcellular compartments formed by three membranes (the outer membrane, cell membrane, and thylakoid membrane), namely, the periplasmatic space, the cytoplasm, and the thylakoid lumen. The MC was found mostly in the thylakoid area through immunogold labeling combined with transmission electron microscopy (TEM)^18^. This technique, however, is limited by two factors: i) preparation of cells using a dehydration series in ethanol makes inspection under noninvasive conditions impossible and ii) only a small percentage of MC molecules are actually labeled due to the processing of the cells. Moreover, even when using improved cryofixation/cryosectioning protocols without dehydration and minimizing dislocation^19,20^, the signal intensity remained low and showed interference with background noise^20^. Immunogold labeling has been further used to quantify MCs in colonies or filaments both under laboratory and field conditions^21^ as well as to visualize MCs in the cell using standard light microscopy^22^.

**Figure 1:**
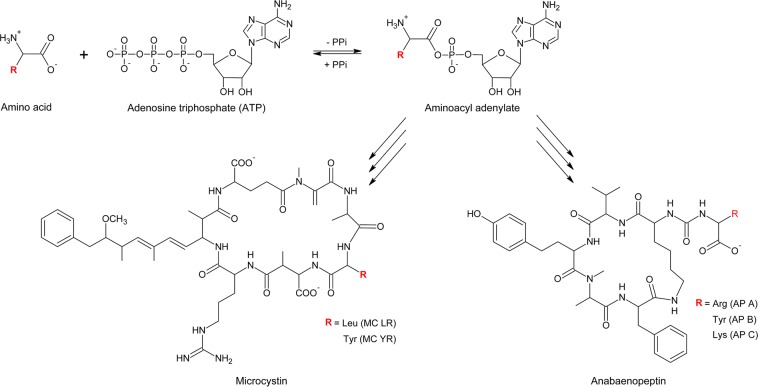
Scheme of variable amino acid adenylation and incorporation into anabaenopeptin (AP) molecule exocyclic pos. 1 or microcystin (MC) molecule pos. 2 in the NRPS pathway by the bispecific (promiscuous) A domain ApnAA_1_ in *Planktothrix agardhii* or McyBA_1_ in *Microcystis aeruginosa*.

We aimed to localize toxic/bioactive peptides using a noninvasive approach by fusing advanced molecule imaging with peptide detection using bio-orthogonal labeling, which employs small molecules that can penetrate the cell membranes directly without treatment and potentially allow for live detection. The genetic basis of such an application builds on the fact that the same cyanobacteria genotypes produce structural variants differing in various amino acids, e.g., *Planktothrix* coproduces MC-RR, MC-LR and MC-HtyR^8,23^, implying coproduction of MC variants carrying Arg, Leu or Hty in pos. 2 of the MC molecule, respectively. Analogously, *Microcystis* cosynthesizes Leu or Tyr in pos. 2 of the MC molecule^7,24^. For the APs, the cooccurrence of various structural variants (AP B, AP F, AP A or Oscillamide (Osc) Y) in isolated genotypes has been observed^9,12^ implying coproduction of AP variants carrying Arg and Tyr in exocyclic pos. 1 of the AP molecule. The A domain of the initiation module of the NRPS, ApnA, was genetically characterized and could be heterologously expressed and used *in vitro* as a model system to biochemically characterize the genetic variation observed within these A domains^9,10^ (Fig. 1).

**Figure 2:**
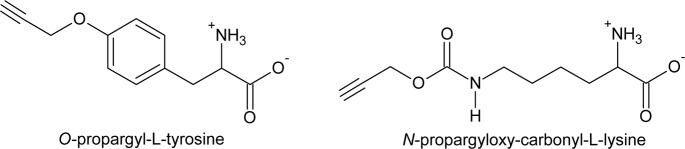
Molecules used for bioorthogonal peptide labeling in *Planktothrix agardhii* and *Microcystis aeruginosa* in this study: O-propargyl-L-tyrosine (Prop-Tyr) and N-propargyloxy-carbonyl-L-lysine (Prop-Lys).

**Figure 3:**
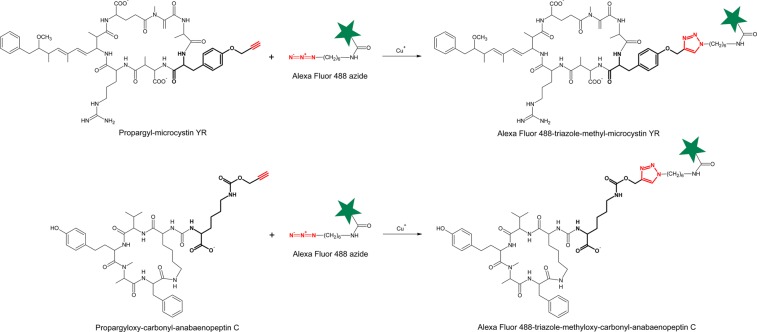
Copper-catalyzed azide alkyne cycloaddition (CuAAC), as exemplified for the detection of anabaenopeptin (AP) or microcystin (MC) molecules. The AP peptides carrying Lys alkyne in exocyclic pos. 1 or MC peptides carrying Tyr alkyne in pos. 2 react with Alexa Fluor 488 azide. The azide/alkyne moieties forming the triazole conjugate are shown in red. Star indicates the chromophore.

Understanding the molecular basis for bispecificity (or even promiscuity) opens the possibility of introducing unnatural amino acids with azido or alkyne groups enabling a subsequent click chemistry reaction^25,26^. This technology might be used to probe the synthesis of AP and MC using copper-catalyzed click chemistry reactions^27^. This biochemical approach uses nonnatural functional groups introduced by metabolic labeling with a precursor, e.g., 4-azidophenylalanine (Az), *N*-propargyloxy-carbonyl-L-lysine (Prop-Lys), *O*-propargyl-L-tyrosine (Prop-Tyr), (Fig. 2), for subsequent labeling of the resulting modified peptide (Fig. 3)^27^. In combination with selective bioorthogonal reactions, such as the strain-promoted azide-alkyne cycloaddition (SPAAC), derivatized secondary metabolites could be also localized directly inside the cell *in vivo*^28–30^. Indeed, the heterologously expressed ApnAA_1_ domain of the wild-type (WT) *Planktothrix* strain PCC 7821 was shown to activate not only Arg and Tyr but also Az^10^, suggesting that activation of this bioorthogonal substrate might occur under natural growth conditions.

In this study, we applied noninvasive labeling of toxic or bioactive peptides (MC and AP) using nonnatural functional groups as precursors (e.g., amino acid-alkynes), followed by subsequent labeling by an azide-modified fluorophore through a copper-catalyzed click chemistry reaction. In addition to chemical-analytical techniques to monitor the generation of modified (clickable) peptides, sensitive *in vivo* techniques such as flow cytometry and laser scanning confocal microscopy were used to qualitatively and quantitatively analyze toxic or bioactive peptide distribution at the intercellular and intracellular levels. Together, these findings showcase a new approach to cyanobacterial physiology and ecology that quantitatively links objectively characterized bioactive metabolites, in particular their synthesis and storage, to the producers at the single cell level.

## Results

### Synthesis of modified peptide structures enabling a subsequent click chemistry reaction

**Figure 4:**
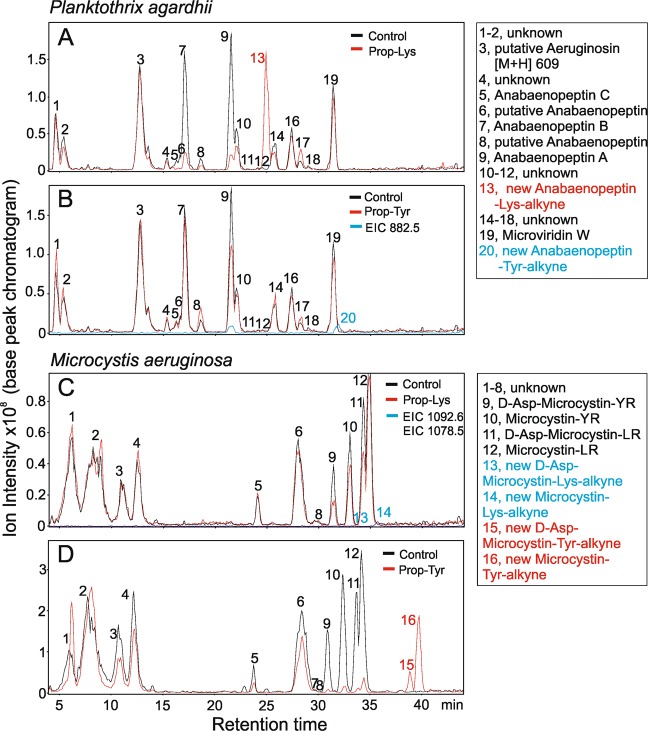
LC-MS chromatograms for *Planktothrix agardhii* No371/1 and *Microcystis aeruginosa* strain Hofbauer showing the synthesis of new AP/MC peptides carrying the alkyne moiety to be used for click chemistry reactions, such as copper-catalyzed azide-alkyne cycloaddition. (**A**,**B**) *Planktothrix agardhii* strain No371/1 synthesizing new AP-Lys-alkyne [M + H]^+^ 891.6 or AP-Tyr-alkyne [M + H]^+^ 882.6; (**C**,**D**) *Microcystis aeruginosa* strain Hofbauer synthesizing new MC-Lys-alkyne [M + H]^+^ 1092.6 or DM-MC-Tyr-alkyne [M + H]^+^ 1069.5 and MC-Tyr-alkyne [M + H]^+^ 1083.5. For a list of protonated masses recorded for all fractions, see Supplemental Tables S1 and S3. Controls from cells grown and processed under identical conditions but without substrate.

Our experiments on *Planktothrix agardhii* strain No371/1, which carries the most promiscuous ApnAA_1_ domain^31^, indicated that the incorporation of either Prop-Lys or Prop-Tyr resulted in new AP peptides carrying the incorporated alkyne moiety (Fig. 4A,B). As a result of Prop-Lys incorporation, a new AP peptide with [M + H]^+^ 891.6 was observed with a high proportion at the expense of natural AP molecule variants such as AP B, AP A and AP C (Table 1, Supplemental Table S1). MS^n^ fragmentation identified that the new AP molecule that arose from the substitution of Arg to Prop-Lys resulted in a new AP alkyne molecule from AP B [M + H]^+^ 837.5 minus Arg (174.2) plus Prop-Lys (228.3) = AP-Lysine alkyne (891.6). Fragments included [M + H]^+^ 637.4 (Lys + Val + Hty + MAla + Phe + 2H), [M + H]^+^ 619.4 (Lys + Val + Hty-H_2_O + MAla + Phe + H), [M + H]^+^ 534.4 (Lys + Val + Hty-H_2_O + Phe + H), [M + H]^+^ 460.3 (Lys + Val + MAla + Phe + 2H), [M + H]^+^ 387.3 (Lys + Val + Hty-H_2_O + H), which were considered indicative of the original AP molecule ring structure (Supplemental Table S2). Analogously, a new AP peptide derived from AP A by substituting Tyr with Prop-Tyr was observed according to the following calculation: AP A (844.5) − Tyr (181.2) + Prop-Tyr (219.2) = AP-Tyr-alkyne (882.6). Again, the MS^n^ fragments indicated an unmodified ring structure of the AP molecule: [M + H]^+^ 637.4, [M + H]^+^ 619.4, [M + H]^+^ 534.4, [M + H]^+^ 460.4, [M + H]^+^ 387.3. Thus, indirect evidence from fragmentation suggested that the alkyne-modified amino acid occurred in exocyclic position 1 of the AP molecule. To investigate the nontargeted incorporation of Prop-Lys or Prop-Tyr, the retention time and protonated mass of the elution fractions from the control and treatments were compared (Supplemental Table 1). With the exception of the modified AP molecules, the retention times and protonated masses were consistent.

**Table 1:** Peptide contents (in percentage of control) of original and modified anabaenopeptin (AP 891, AP 882) and microcystin peptide (MC 1069, MC 1078, MC 1083, MC 1092) structural variants detected in *Planktothrix agardhii* strain No371/1 and in *Microcystis aeruginosa* strain Hofbauer fed with nonnatural amino acids (Prop-Tyr, *O*-propargyl-L-tyrosine; Prop-Lys, *N*-propargyloxy-carbonyl-L-lysineN-propargyloxy-carbonyl-L-lysine). Controls represent cells grown and processed under identical conditions but without substrate (three biological replicates). Controls were set arbitrarily to 100% and contained 394 ± 167 (SE) ng of AP B equivalents or 632 ± 184 ng of MC-LR equivalents per mg dry weight.

Peptide	Retention time (min)	Control	Prop-Lys	Prop-Tyr
***Planktothrix agardhii*** **No371/1**				
AP C [M + H]^+^ 809	16.4	2.6 ± 0.5	0.07 ± 0.07	1.7 ± 1
AP B [M + H]^+^ 837	17.3	52 ± 5	6 ± 1	25 ± 11
AP A [M + H]^+^ 844	21.6	45 ± 5	2.1 ± 0.9	13 ± 6
AP [M + H]^+^ 891	25.1	n.d.	48 ± 4	n.d.
AP [M + H]^+^ 882	31.9	n.d.	n.d.	1.4 ± 0.7
AP total		100	56 ± 6	42 ± 18
***Microcystis aeruginosa*** **Hofbauer**				
Asp-MC YR [M + H]^+^ 1031	31.2	13 ± 1.4	5 ± 2	0.2 ± 0.1
MC-YR [M + H]^+^ 1045	32.8	23 ± 2	12 ± 2	0.9 ± 0.1
Asp-MC LR [M + H]^+^ 981	34.2	22 ± 2	13 ± 5	0.7 ± 0.2
MC-LR [M + H]^+^ 995	34.9	42 ± 3	28 ± 6	1.7 ± 0.4
MC [M + H]^+^ 1078	34.3	n.d.	0.2 ± 0.2	n.d.
MC [M + H]^+^ 1092	35.6	n.d.	0.7 ± 0.3	n.d.
MC [M + H]^+^ 1069	39.4	n.d.	n.d.	4.3 ± 1.8
MC [M + H]^+^ 1083	40.3	n.d.	n.d.	14 ± 4
MC total		100	58 ± 15	22 ± 5

n.d., not detected.

Analogously, *Microcystis aeruginosa* strain Hofbauer coproducing (de)methylated-MC-YR and (de)methylated(DM)-MC-LR^7,24^ was fed with Prop-Lys and Prop-Tyr under the same conditions and analyzed for new MC structural variants carrying the alkyne moiety (Supplemental Table S3). Feeding with Prop-Lys resulted in two new MC-structural variants, as predicted from the calculation (DM-) MC-LR [M + H]^+^ 995.5 minus Leu (131.2) plus Prop-Lys (228.3) = MC-Lys-alkyne [M + H]^+^ 1092.6 or 1078.5 (DM-MC-Lys-alkyne). Feeding with Prop-Tyr resulted in two new MC-structural variants, as predicted from the following calculation: DM-MC-YR [M + H]^+^ 1031.5 minus Tyr (181.2) plus Prop-Tyr (219.2) = DM-MC-Tyr-alkyne [M + H]^+^ 1069.6 and MC-YR [M + H]^+^ 1045.5 minus Tyr (181.2) plus Prop-Tyr (219.2) = MC-Tyr-alkyne [M + H]^+^ 1083.5 (Fig. 4C,D). For all MC variants, the MS spectra revealed the characteristic fragment of the conserved Adda moiety (2S, 3S, 8S, 9S)-3-amino-9-methoxy-2,6,8-trimethyl-10-phenyldeca-4,6-dienoic acid), [M + H]^+^ 135 and the theoretical [M + H]^+^ value of the corresponding fragmented MC molecule. Other characteristic MS^n^ fragments included [M + H]^+^ 599.4 (Arg + Adda + Glu + H) and [M + H]^+^ 155.0 (Mdha + Ala + H), Supplemental Table S4. Thus, analogous to AP synthesis, the expected modified MC-molecule carrying the alkyne moiety was found, and fragmentation indirectly indicated the occurrence of the alkyne-modified amino acid within the ring structure of the MC molecule. Additionally, for *M. aeruginosa*, according to the inspection of the respective LC-MS chromatograms, the elution fractions showed consistent masses, suggesting little nontargeted peptide modification.

### Quantification of click chemistry-labeled peptides in cells

**Figure 5:**
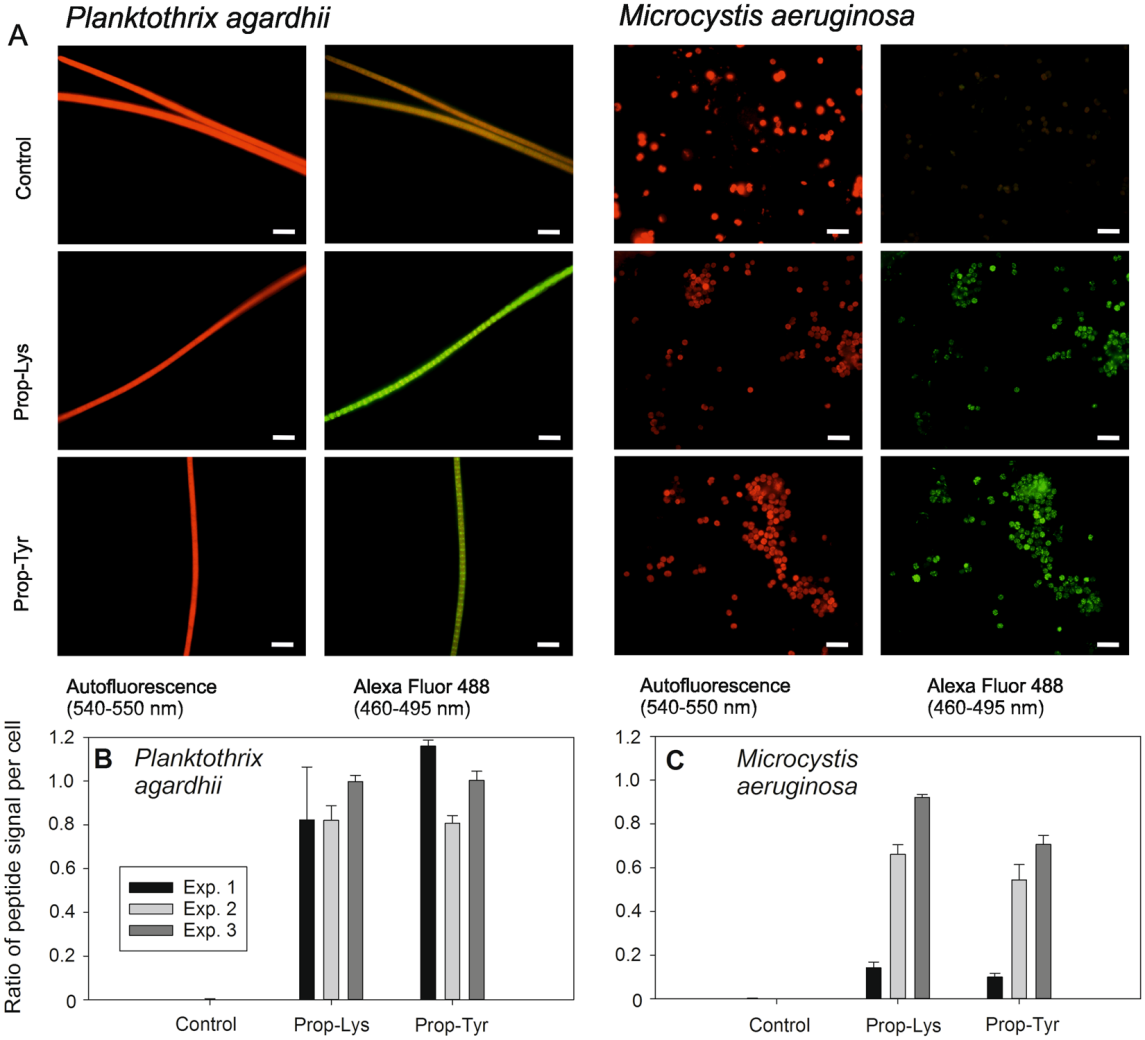
(**A**) Microphotographs of *Planktothrix agardhii* No371/1 and *Microcystis aeruginosa* recorded under two emission spectra (autofluorescence and Alexa Fluor 488) by epifluorescence microscopy (100-fold magnification). Bar is 10 µm. Controls (cells grown and processed under identical conditions but without substrate); Prop-Lys, cells fed with Prop-Lys; Prop-Tyr, cells fed with Prop-Tyr, and used for bioorthogonal labeling. (**B**,**C**) Percentage of peptide labeling signals in cells as recorded by epifluorescence microscopy (different shades of gray represent three biological replicates). (**B**) *Planktothrix agardhii* No371/1 synthesizing new AP-Lys-alkyne [M + H]^+^ 891.6 or new AP-Tyr-alkyne [M + H]^+^ 882.6; (**C**) *Microcystis aeruginosa* strain Hofbauer synthesizing new MC-Lys-alkyne [M + H]^+^ 1092.6 or DM-MC-Prop-Tyr-alkyne [M + H]^+^ 1069.5 and MC-Prop-Tyr-alkyne [M + H]^+^ 1083.5.

Cells were incubated and prepared for the click chemistry reaction by copper-catalyzed triazole formation from an azide and an alkyne used interchangeably (see above), i.e., for *P. agardhii* and *M. aeruginosa* previously grown with Prop-Lys and Prop-Tyr, and the Alexa Fluor 488 Az chromophore was used. Cells were counted by epifluorescence microscopy (Olympus BX53) using the exposure time automatically adjusted for autofluorescence (AF) and applying the same exposure time to Alexa Fluor 488 emission for visualizing the peptide signal. Filaments or cells from controls (i.e., cells grown and processed under identical conditions but without substrate) showed typical AF but much less fluorescence using the Alexa Fluor 488 filter (Fig. 5A). In contrast, for the Prop-Lys and Prop-Tyr treatments, the observed signal strength for AF and Alexa Fluor 488 was comparable. However, while the AF signal was always homogeneously distributed across the cells, the Alexa Fluor 488 signal appeared more heterogeneous. The filaments or cells were counted quantitatively, which showed that peptide-labeled cells were frequent for *P. agardhii* strain No371/1 fed both Prop-Lys and Prop-Tyr fed cells (Fig. 5B), although unstained filaments were still observed. In general, the length of the *Planktothrix* filaments was not significantly reduced by feeding with nonnatural amino acids (Table 2). Similarly, for *M. aeruginosa*, the proportion of stained Prop-Tyr-fed cells was found to be significantly increased, though unlabeled cells were also present (Fig. 5C). Together, these results provide evidence that peptides were labeled in the cells and that the peptide signal was separated from the natural AF. Filaments or cells observed from the control showed signals for a few individual cells only, which implied unspecific labeling but interfered with the counting of the peptide signals to a lesser extent.

**Table 2:** Growth rates and peptide signals in *Planktothrix* filaments and *Microcystis* cells fed with nonnatural amino acids (Prop-Tyr, *O*-propargyl-L-tyrosine; Prop-Lys, *N*-propargyloxy-carbonyl-L-lysine) as observed by epifluorescence microscopy. Controls represent cells grown and processed under identical conditions but without substrate (three biological replicates).

	Growth rate (day^−1^)	No filaments or cells	Filament length in total (in µm)	(mean ± SE) filament length in µm^a^	Percentage of non labeled filaments or cells	Ratio of peptide signal per cell^a^			
						min	max	average ± SE	P-value^b^
***Planktothrix agardhii*** **No371/1**									
Control	0.28 ± 0.03	50; 106; 20	2809; 1637; 1868	50 ± 3; 15 ± 3; 93 ± 3	100; 99; 100	0; 0; 0	0; 0.3; 0	0 ± 0; 0.003 ± 0.003; 0 ± 0	
Prop-Lys	0.28 ± 0.03	11; 36; 22	493; 608; 2586	45 ± 4; 17 ± 1; 118 ± 8	36; 3; 0	0; 0; 0.8	2.4; 2.1; 1.3	0.82 ± 0.24; 0.82 ± 0.07; 1.0 ± 0.3	<0.001
Prop-Tyr	0.28 ± 0.03	22; 81; 20	1118; 1114; 1869	51 ± 5; 13 ± 1; 94 ± 8	0; 4; 0	0.9; 0; 0.6	1.5; 1.5; 1.3	1.2 ± 0.02; 0.81 ± 0.04; 1.0 ± 0.04	<0.001
***Microcystis aeruginosa*** **Hofbauer**									
Control	0.35 ± 0.04	431; 451; 514	n/a	n/a	99.8; 100; 100	0; 0; 0	0.03; 0; 0	0.002 ± 0.002; 0 ± 0; 0 ± 0	
Prop-Lys	0.34 ± 0.02	259; 913; 581	n/a	n/a	86; 34; 9	0.06; 0.31; 0.78	0.36; 1.0; 1.0	0.14 ± 0.03; 0.66 ± 0.04; 0.92 ± 0.01	<0.001
Prop-Tyr	0.36 ± 0.04	522; 608; 735	n/a	n/a	92; 30; 42	0; 0.13; 0.08	0.27; 1.0; 1.0	0.1 ± 0.01; 0.54 ± 0.07; 0.71 ± 0.04	<0.001

^a^Filaments were measured in length and signals per unit of length were recorded.

^b^Peptide signals were compared between controls and treatments using nonparametric Mann Whitney Rank Sum test.

n/a, not applicable.

**Figure 6:**
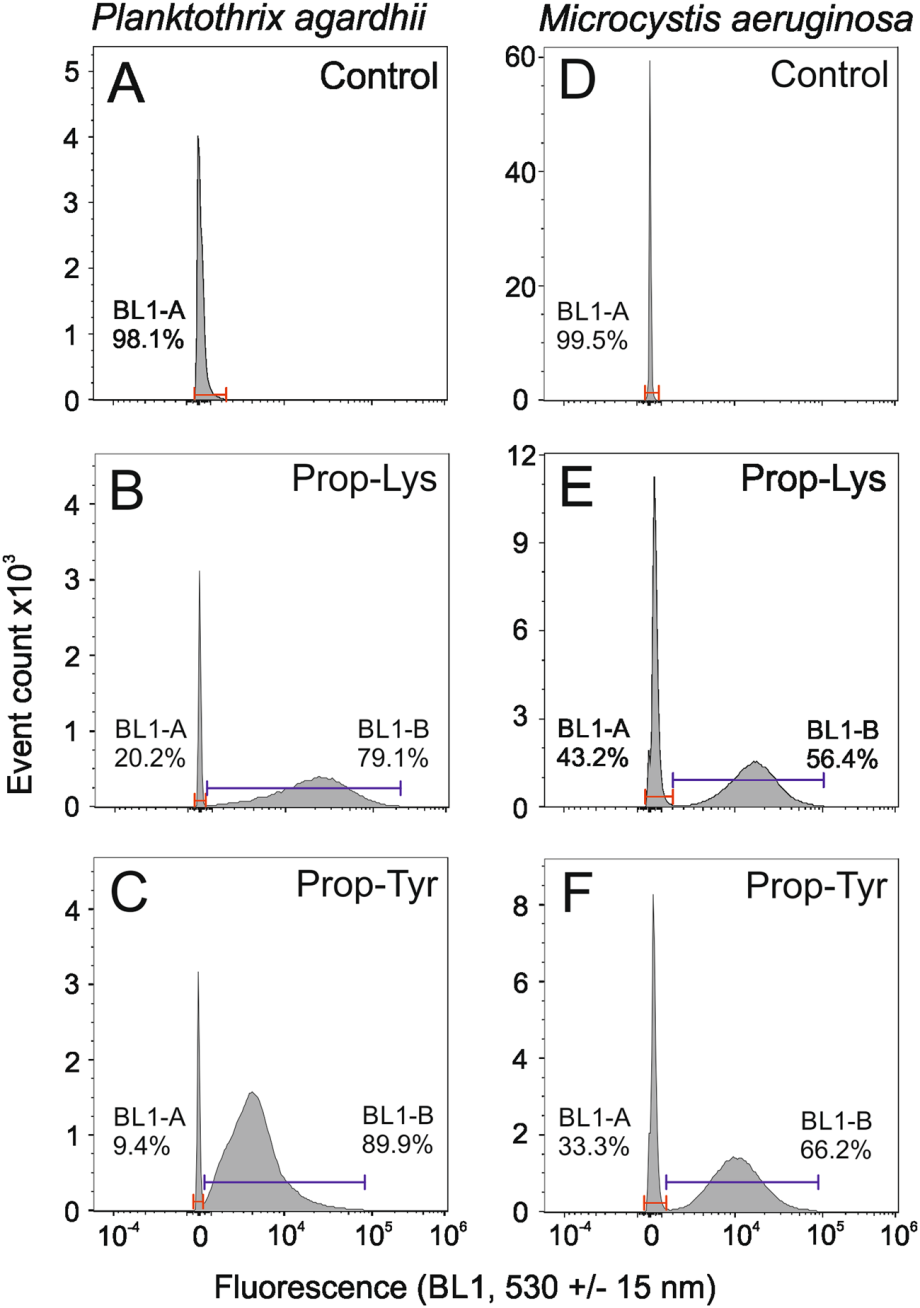
Flow cytometry-based detection of (**A**–**C**) *Planktothrix agardhii* No371/1 cells carrying labeled AP peptides resulting from Prop-Lys or Prop-Tyr and (**D**–**F**) *M. aeruginosa* strain Hofbauer cells carrying labeled AP peptides resulting from Prop-Lys or Prop-Tyr stained using Alexa Fluor 488 alkyne compared with the control. Controls represent cells grown and processed for click chemistry under identical conditions but without substrate. Different colors through the indicated gates mark unlabeled cells (as inferred from controls, BL1-A) or labeled cells (BL1-B) and the respective proportion is indicated. One parallel is shown, and the other two parallels are shown in Supplemental Figs. 1 and 2.

Finally, by means of flow cytometry, a much higher number of filaments or cells carrying the labeled peptides could be counted and discriminated from unlabeled cells or filaments (Fig. 6, Supplemental Figs. 1 and 2). According to the microscopic counting results, labeled and unlabeled filaments or cells cooccurred. The unlabeled cells were distinguished as a sharp fraction because they were not stained using the peptide Alexa Fluor 488 signal. In contrast, the labeled cells showed a higher variability of fluorescence intensity by several orders of magnitude. For *P. agardhii*, on average, cell proportions of 77% ± 3 (1 SE) and 75% ± 7% were found labeled for Prop-Lys and Prop-Lys, respectively. Similarly, on average, 75 ± 19% and 84 ± 9% of the cells were labeled for *M. aeruginosa*. It was concluded that the peptide-labeled cell fractions were reliably differentiated from the unlabeled controls, while the influence of unspecific labeling and/or autofluorescence was found to be of minor importance.

### Subcellular localization of toxic or bioactive peptides

**Figure 7:**
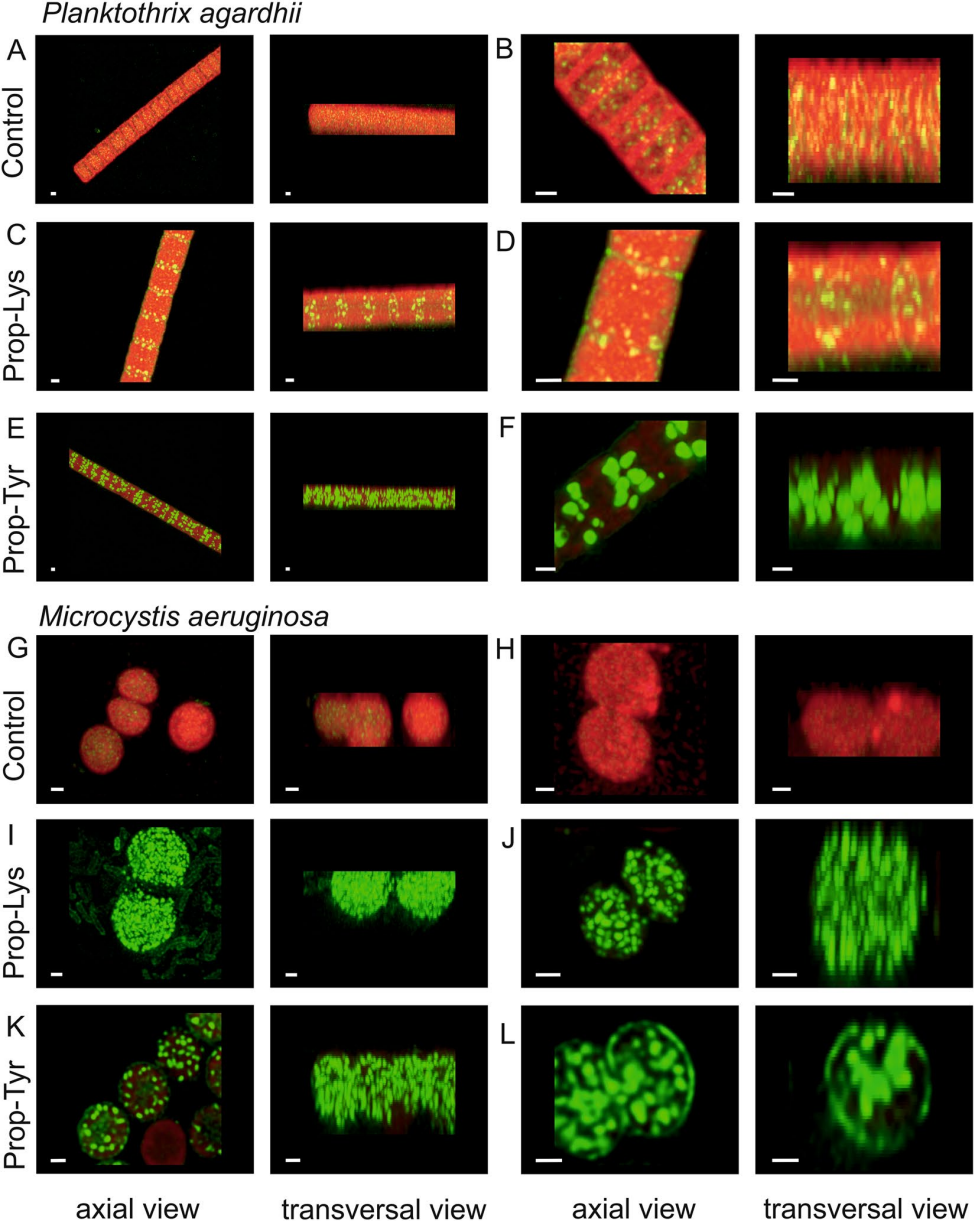
High-resolution fluorescence images from cells with labeled AP/MC peptides from cyanobacterial cultures as analyzed by LC-MS in Fig. 4. (**A**–**H**), *Planktothrix agardhii* No371/1 cells from (**A**,**B**) controls (cells grown and processed under identical conditions but without substrate); (**C**,**D**) cells synthesizing new AP-Lys-alkyne [M + H]^+^ 891.6, (**E**,**F**) cells synthesizing new AP-Tyr-alkyne [M + H]^+^ 882.6. (**G**–**L**) *Microcystis aeruginosa* strain Hofbauer cells from (**G**,**H**) controls; (**I**,**J**) cells synthesizing new MC-Prop-Lys-alkyne [M + H]^+^ 1092.6; (**K**,**L**) cells synthesizing new DM-MC-Prop-Tyr-alkyne [M + H]^+^ 1069.5 and MC-Prop-Tyr-alkyne [M + H]^+^ 1083.5. Excitation at 498 (Alexa Fluor 488) and 620 nm (AF) by a white light laser using an SP8 laser scanning microscope (Leica). Z-stacks were obtained at depth intervals of 0.15 µm, resulting in ca. 40 slices per cell. The scale bar denotes one µm. Images were deconvoluted using Huygens Software (Scientific Volume Imaging (SVI), VB Hilversum, The Netherlands, http://svi.nl)).

Based on epifluorescence imaging, the green Alexa Fluor 488 labeled peptides appeared as rather distinct entities inside the cell and showed a cell-wide distribution (Fig. 5A,B). The same slides inspected by epifluorescence microscopy were subjected to high resolution microscopy using an LSM SP8 microscope with a resolution below one micrometer. For *Planktothrix*, the AF signal observed was relatively homogeneous throughout the entire cell. In contrast, both the various AP-peptide signals and the MC-peptide signal occurred in distinct entities inside the cells and did not show a more homogeneous distribution, as observed for the AF signal (Fig. 7). For individual cells for each channel, total signal intensities were calculated, and the ratio of the green peptide signal vs AF and the maximum intensity were compared between treatments. For *Planktothrix*, this ratio ranged on average between 2.2 ± 0.5 (SE), 0.5 ± 0.1 and 0.09 ± 0 for Prop-Tyr, Prop-Lys, and controls, respectively (p = 0.002, n = 14). Similarly, for *Microcystis*, the ratio varied from 4.1 ± 1.4, 1.5 ± 1, and 0.06 ± 0.02 for Prop-Tyr, Prop-Lys, and controls, respectively (p = 0.04, n = 9) (Supplemental Fig. 3A–C). Not surprisingly, the maximum peptide signal intensities differed between treatments for both genera *Planktothrix* (p = 0.001) and *Microcystis* (p = 0.016), while the maximum AF intensities remained unaffected (p = 0.227 or p = 0.371). However, colocalization coefficients decreased for both *Planktothrix* (p = 0.003) and *Microcystis* (p = 0.09) as a result of AP or MC peptide labeling, Supplemental Fig. 3D implying that peptide signals were not physically related to thylakoids and the light harvesting antennae systems responsible for the observed AF. To exclude the possibility that the formed entities were artifacts due to the cell fixation using paraformaldehyde (PFA) procedure, aliquots of cells were not fixed but treated in parallel for the click chemistry reaction. The same distinct entities and intracellular cell-wide distribution were observed (Supplemental Fig. 4), implying that the chromophore Alexa Fluor 488 azide diffuses into the cells even without prior fixation and membrane treatment and that the fixation procedure was not related to the observed peptide entities.

### Visualization of cellular compartments using standard immunofluorescence

The observation that peptide signals occurred as distinct entities rather than homogeneously across the cell volume was further analyzed by visualizing cellular compartments using standard immunofluorescence (IF). Three following primary antibodies were used for detection: RbcL (Rubisco large subunit, form I and form II) for the cytoplasm of cyanobacteria, PsbA (D1 protein of PSII, C-terminal) as a thylakoid membrane marker and FtsZ (prokaryotic cell division GTPase) for the cytoplasm. To facilitate binding of primary antibodies to the three organelles, cells were harvested as described above but fixed with 96% ethanol (EtOH, −20 °C) for 15 min, washed and treated for immunolocalization according to standard protocols. Using a secondary antibody conjugated to the Alexa Fluor 488 chromophore revealed cellular compartments, i.e., RbcL occurred in more distinct entities that are most likely associated with carboxysomes, whereas PsbA was distributed across the cell diameter. FtsZ was distributed across the cell or formed a ring structure in the putative cell division plane (Supplemental Fig. 5). To document an (unwanted) extraction of peptides by the EtOH fixation procedure, signal intensities of peptides vs AF were calculated for individual cells (n = 20) for both treatments (Prop-Lys, Prop-Tyr) as well as the IF staining procedure and compared with signal intensities obtained from PFA-fixed cells. For *P. agardhii*, the EtOH fixation procedure led to a significant loss of labeled AP, as indicated by a drop in signal intensity as well as in the ratio of peptide signal intensity to AF intensity (Supplemental Fig. 6A–C). This decrease in AP signal intensity was independent of treatment (Prop-Lys, Prop-Tyr) or the use of antibody and rather occurred because of the general fragmentation of filaments into individual cells due to EtOH fixation (e.g., Supplemental Fig. 5). However, for *M. aeruginosa*, no such loss of labeled MC was observed, and cells fixed in EtOH showed rather stable signal intensities when compared with cells fixed in PFA only (Supplemental Fig. 7A–C). Consequently, only the *M. aeruginosa* colocalization results for both chemically labeled MC and immunostained proteins were quantified and statistically compared.

**Figure 8:**
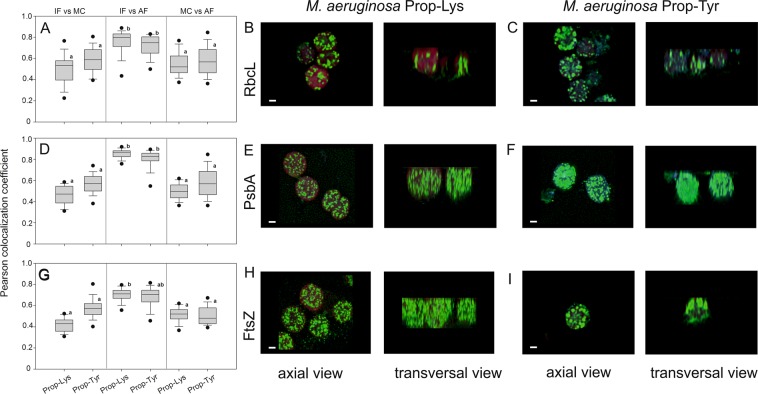
Pearson colocalization coefficients calculated in pairs (immunofluorescence (IF) vs. labeled MC, IF vs. autofluorescence (AF) signals and labeled MC vs. AF) for three proteins: (**A**–**C**) RbcL, (**D**–**F**) PsbA, and (**G**–**I**) FtsZ, indicating either cytoplasm (RbcL, FtsZ) or thylakoid membranes (PsbA). The scale bar denotes one µm. Pearson colocalization coefficients were calculated using Huygens software. Letters indicate subgroups not significantly different at p < 0.05 if an overall difference was found (n = 20).

For all three proteins, RbcL, PsbA and FtsZ, the correlation between pixels obtained from IF and AF was highest and differed significantly from the (lower) correlation measurements calculated with the clicked MC signals (Fig. 8). The correlation measurements for PsbA and AF (median 0.867 and 0.827 for Prop-Lys and Prop-Tyr, respectively) were significantly higher when compared with RbcL vs AF (0.797, 0.750) and FtsZ vs AF (0.711, 0.706), (p < 0.001), which was probably because of the location of PsbA within thylakoid membranes. On the other hand, for all three protein immuno stainings, the correlation between labeled MC and AF signals was significantly lower, i.e., PsbA (median 0.498, 0.623), RbcL (0.521, 0.569), FtsZ (0.52, 0.48), albeit positively related. In addition, for all three protein immuno stainings, relatively low, albeit positive, relationships between IF and labeled MC were observed, i.e., PsbA (median 0.471, 0.57), RbcL (0.534, 0.589), and FtsZ (0.429, 0.573) did not differ between labeled MC and AF pairs.

In summary, positive relationships in cellular distribution between labeled MC and various cellular compartments, as indicated by protein localization as well as AF, were regularly observed. However, the correlation indices between proteins and AF were even higher, implying that the compartments of RbcL, PsbA, and FtsZ were related to the distribution of labeled MC in the cell but only to a minor extent. In other words, the overall decrease in colocalization coefficients between labeled MC and all tested compartments corresponds to the formation of distinct entities described above.

## Discussion

### Methodology and application of toxic or bioactive peptide labeling

Methods for labeling and visualizing toxic peptides such as MC in cyanobacteria have been sought for a long time. This study takes advantage of promiscuous enzyme domains that are part of the NRPS pathway. Those enzyme domains have been characterized in isolated clonal strains with regard to the corresponding peptide structural variability, leading to the selection of genotypes carrying A domains suitable for the incorporation of unnatural amino acids enabling a standard click chemistry approach. Unnatural amino acids are fed at millimolar amounts during logarithmic growth culture conditions, and cells are processed by applying standard copper-catalyzed azide alkyne cycloaddition (CuAAC) for click chemistry detection. In contrast to the antibody-based technique using fixation and perforation of the cell wall, the bioorthogonal labeling technique benefits from small molecules that can penetrate the cell membranes directly without treatment, which potentially allows for live detection.

Not all AP- or MC-producing genotypes carry unspecific (promiscuous) adenylation domains such as ApnAA_1_ or McyBA_1_ that would exclude the incorporation of the unnatural amino acids required for bioorthogonal labeling. Thus, under the current protocol, certain (specified) genotypes will be precluded from candidates for subcellular labeling of toxic or bioactive peptides because of their genetic basis. However, follow-up investigations could possibly identify potential unnatural amino acids that are also integrated by more specific A domain genotypes. On the other hand, nontargeted incorporation of unnatural amino acids cannot be excluded for all genotypes but requires monitoring as has been applied in this study.

One possible field of application might include monitoring the production of toxic/bioactive peptides in mass culture (e.g., by *in situ* fluorescence measurements). This could be performed using a standard cell fixation protocol as used in this study or even observing living cells. For this aim, the reagent would need to pass the cell membrane of living cells without treatment^32^. Indeed, pilot tests using copper-catalyzed triazole formation as described above but without a fixation procedure using PFA and Triton showed that this is possible in principle (Supplemental Fig. 4). There is continuous progress in the improvement of toxic/bioactive peptide production for biotechnological applications, i.e., through heterologous expression in a genetically engineered cyanobacterium^33^ or in *E. coli*^34^. It is known that the production of a certain MC or AP is physiologically regulated and can even be eliminated by, e.g., genetic mobile elements^35^. Therefore, observation of toxic/bioactive peptide production at the single cell level will enable close supervision and potentially increase the efficiency of the production process.

A second possible field of application might be related to the fate of AP or MC in water, which can be analyzed by fluorescence. According to the observed retention times in LC-MS chromatograms (Fig. 4), the modified peptides carrying the alkyne moiety became more lipophilic but still eluted earlier than naturally occurring lipophilic MC-LW or MC-LF^36^. Since labeled AP or MC peptides can be detected in cells using standard flow cytometry techniques, this invention might lead to a high-throughput technique that could, for example, determine the fate of extracellular peptides and the uptake of labeled peptides by other organisms. In a similar vein, the production of modified MC has been proposed as an anchor group to monitor the delivery of certain drugs during cancer treatment^37^. Recently, the synthesis of an alkyne-labeled MC-LF structural variant, which was suggested to be used for toxin derivatization by click chemistry with an azide-containing reporter molecule, has been reported^38^. The propargylated derivative showed an IC_50_ = 1.7 nM for Protein phosphatase 1 inhibition, suggesting that the natural inhibitory activity was not disturbed. Thus, potential interaction partners might be identified *in vivo* with high-throughput techniques such as flow cytometry.

### Subcellular compartmentation of toxic or bioactive peptides

It has been speculated that the hydrophobic Adda moiety of the MC molecule is bound to membranes, leaving the polar peptide ring free in the cytoplasm^19,39^. Indeed, a structural MC variant, namely, nodularin, has been reported to form pores in lipid bilayers and in native membranes^40^. According to immunogold labeling, the majority of MC molecules have been detected in the thylakoid area^18,19^. Proteomic analysis showed a chemical linkage between immunologically detected MCs and abundant proteins such as phycobilins and Rubisco^17^. Previous research has reported that a significant amount of MC is conjugated via the free methylene group of the N-methyl-dehydroalanine (Mdha) moiety in pos. 7 to a free cysteine residue of proteins such as the most abundant phycobiliproteins^17,41^. On the other hand, the AP molecules carrying N-methylated groups at pos. 5 (Hty or Ala) of the AP molecule may not be able to undergo analogous chemical thiolation reactions under intracellular neutral pH conditions^42^. While chemical binding for AP molecules has not been directly investigated, chemical thiolation reactions for MC variants lacking the N-methyl-dehydroalanine (Mdha) or dehydroalanine (Dha) moiety in pos. 7 of the MC molecule has been monitored and found to react much slower under neutral pH conditions^42^. Therefore, in this study, for AP molecules, a more homogeneous distribution of the peptide signal resulting from dissolved AP molecules in the cytosol would have been expected.

Since thylakoid membranes carrying phycobilins can be monitored independently through AF from the peptide labeling signal, the general hypothesis of MC binding to abundant organelles such as thylakoids has been investigated directly by imaging and colocalization analysis of the Alexa Fluor 488 peptide signal (Ex 495) with the AF signal (Ex 620 nm). Furthermore, if AP or MC peptides are located in the cytoplasm or in the periplasmic space, high microscopic resolution by 3D z-stack generation would have qualitatively and quantitatively revealed the distribution along the transection (e.g. Fig. 7). However, rather the opposite was observed; pairwise colocalization coefficients distinctly declined for MC or AP vs AF, implying the localization of peptide signals in certain entities that seem less related to thylakoids and light harvesting antennae systems (Supplemental Fig. 4). Correspondingly, both MC and AP peptide signals appeared as individual entities distributed through the cell, pointing to potential (micro) compartmentation. To better analyze the lack of correlation with thylakoids, three different organelles were immune labeled and colocalized with labeled MC. Since only the MC molecules could be quantitatively recovered after the double staining protocol, the distribution of chemically labeled AP peptides was not analyzed further. However, it is interesting to note that the modified MC molecules all showed lower correlation coefficients not only with AF but also with RbcL, PsbA and FtsZ. Currently, those decreased correlation coefficients can be best explained by the major part of MC occurring in distinct entities, with only a minor part spatially related to the investigated organelles.

As an alternative approach to labeling the MC molecule itself, fluorescence-based *in situ* hybridization (FISH) using a DNA probe covalently attached to an enzyme, such as horseradish peroxidase (HSP), has been applied to distinguish MC producers from nonproducers by epifluorescence microscopy^43^. Furthermore, improved protocols such as tyramide signal amplification (TSA)-FISH have been used to detect the *mcy*A mRNA gene in *M. aeruginosa*^44^ and in *P. agardhii*^45^. Both studies report a nonuniform labeling of *mcy*A mRNA gene expression and suggest an unequal ribosomal distribution as a possible cause. Since the enzymatic reaction of HSP resulting in fluorescence emission cannot be compared directly to the fluorophore signals analyzed in this study, further speculation seems premature. Recently the occurrence of so-called “spots” attributed to MC-LR was described in *M. aeruginosa* strain PCC7806 using the IF technique^46^. In the future, it will be important to determine whether MC and AP are produced in a specific compartment in the cell and/or are collected in some kind of inclusion body, implying an intracellular storage pool. The latter mechanism would help to explain the relatively high intracellular concentration.

In conclusion, the technique used in this study enables linking toxin or bioactive peptide synthesis and potential storage at a single cell or subcellular level. The results of this study are the first to provide evidence for distinct entities of peptides rather than homogeneous distribution across the cell, which raises the question of the mechanism of particular cyanotoxin storage in the cell. Additionally, this technique has the potential to more broadly map how bioactive peptides, especially cyanotoxins, change over space and time at a subcellular level. Given that high resolution at the phenotype level is genetically linked to the producer organism, these data should better elucidate the processes that drive cyanobacterial toxin production in nature.

## Materials and Methods

### Study organisms

Based on the variability in substrate selection of A domains^7,31^, one strain of *Planktothrix* and one strain of *Microcystis* were selected and tested for incorporation of unnatural amino acids to be subsequently used for bioorthogonal labeling in the cells. *P. agardhii* strain No371/1 (isolated from Moose Lake, Alberta, Canada, by Rainer Kurmayer in 2005) has been reported to carry a promiscuous ApnAA_1_ domain^31^, resulting in diverse coproduced AP structural variants with different amino acids in exocyclic pos. 1, i.e., combinations of Arg, Tyr, Lys and unknown amino acids. Phylogenetic analysis of the corresponding *apn*AA_1_ gene locus (1702 bp) revealed that this strain belonged to phylogenetic clade D, GeneBank accession number KU639999, showing the most diverse substrate activation (Fig. 6 in^31^). For labeling MC structural variants, *M. aeruginosa* strain Hofbauer (isolated from Lake Neusiedl by Barbara Hofbauer, Austria, in 1982), coproducing (de)methylated-MC-YR and (de)methylated- MC-LR, was used^47^. The corresponding *mcy*BA_1_ gene locus (1312 bp) was assigned to the phylogenetic clade named *mcy*B_1_(B), GenBank accession number MN935899, which was not affected by a domain-wide recombination event^7,48^.

### Growth conditions

To investigate strain-specific variation in MC and AP production and localization, nonaxenic strains were grown in BG11 medium^49^ under sterile conditions at 20 °C and 50 µE m^−2^ s^−1^ semicontinuously following the turbidostat principle^50^. Unnatural amino acid feeding experiments were repeated two times. The growth of the cells was monitored by measuring the absorbance at 600 nm (1 cm light path) twice a week (40 ml volume), and each time OD_600_ ≥ 0.1, the culture was diluted down to OD_600_ = 0.01. For *P. agardhii *strain No371/1, the number of filaments was found to be linearly related to the OD according to y = 7E-06 x + 0.0513 (R^2^ = 0.76), where y is the OD_600_ and x is the number of filaments/ml (n = 13). Accordingly, OD = 0.01 was equal to 662 ± 265 filaments/ml (equivalent to 0.005 ± 0.002 mm^3^/ml or 0.01 ± 0.001 mg DW/ml, n = 3). For *M. aeruginosa* strain Hofbauer, the number of cells at OD = 0.01 was 62866 ± 29059 (equal to 0.002 ± 0.001 mm^3^/ml or 0.002 ± 0.0004 mg DW/ml).

### Feeding of unnatural amino acids

Cyanobacteria were grown with 0.05 mM unnatural amino acid substrates *N*-propargyloxy-carbonyl-L-lysine (Prop-Lys), molecular weight 228.25, (SC-8002, Sirius Fine Chemicals SiChem GmbH, Bremen, Germany), and *O*-propargyl-L-tyrosine (Prop-Tyr), molecular weight 219.24, (HAA1971, Iris Biotech GmbH, Marktredwitz, Germany), which were dissolved in 1 mM NaOH and added to the BG11 medium at the time of inoculation (Fig. 2). According to a pilot experiment that was performed for a period of 11 days, concentrations above 0.05 mM unnatural amino acid substrates negatively affected growth (data not shown). Furthermore, under the specified culture conditions, new-modified AP or MC peptides showed a notable increase after 3–6 days in batch culture, and a growth period of 6 days was used in all subsequent feeding experiments.

### Cell harvesting and peptide extraction

Cells were harvested by vacuum filtration onto GF/C filters, dried using a vacuum centrifuge and stored at −20 °C. Cells were extracted in aqueous methanol (50/50, v/v) by shaking on ice as described previously^12^, and the peptide extract was purified from cell debris by centrifugation. Peptide structural variants were separated by HPLC (HP 1100, Agilent) using a linear water/acetonitrile (0.05% trifluoroacetic acid) gradient from 80:20 to 50:50 in 45 min at a flow rate of 1 ml min^−1^ in a 30 °C oven, LiChrospher 100 octyldecyl silane (ODS) (5 µm particle size) and LiChroCART 250-4 cartridge system (Merck, Darmstadt, Germany), as described previously^12^. The HPLC system was coupled to an electrospray ionization (ESI) mass spectrometer ion trap (amaZonSL, Bruker) operating in positive ion mode. Nitrogen was used as sheath gas (43 psi, 8 L/min, 300 °C), and helium was used as auxiliary gas. The capillary voltage was set to 5 kV. Under these conditions, peptide structural variants were assigned according to the protonated mass in positive mode (accuracy = 0.15 Da), retention time and specific fragmentation using the enhanced resolution mode (full width at half maximum (FWHM) = 0.35, 50–2000 Da), (MS^1^, MS^2^, MS^3^), (Supplemental Table S1–S4). At MS^2^, the automated fragmentation mode selected the two most intensive mass peaks of MS^1^, while only one major peak of MS^2^ was fragmented under MS^3^ conditions. To determine the potential detection limit, purified AP B (prepared by Judith Blom, Univ. of Zürich, Switzerland) and MC-YR and MC-LR standards (Cyanobiotech GmbH, Berlin, Germany) were used. Injection tests with AP B [M + H]^+^ 837, MC-YR [M + H]^+^ and MC-LR [M + H]^+^ 995 revealed a limit of detection (LOD) of 1 ng (amount injected). To characterize the total peptide content, all peptides were quantified in equivalents of either AP B or MC-LR. According to the linear regression curves y = 1 × 10^7^x + 1 × 10^8^ and y = 2 × 10^7^ x −4 × 10^8^, y is the integrated area in the base peak chromatogram (BPC), and x is ng of AP B (1–270 ng) or MC-LR (1–180 ng) injected. Because of the general difficulty of standardizing molecule detection, the original and modified peptide contents were compared as a percentage of the controls grown without substrate (arbitrarily set to 100%).

### Bioorthogonal labeling

Cell cultures were harvested after 6 days by using the hammer, cork, and bottle method^51^ to destroy the gas vesicles and enable sedimentation during centrifugation (14 000 × g, RT). In general, cells were fixed using standard protocols, such as by 2% paraformaldehyde (PFA), permeabilized using 0.1% Triton and washed with phosphate-buffered saline (PBS). In particular, cells were centrifuged and washed in PBS (3×), fixed in 2% PFA (15 min), washed with PBS (3×), incubated with 0.1% Triton (10 min, RT), washed with PBS (3×) and stored at 4 °C. Cells were subsequently used for the click chemistry reaction (Thermo Fisher Scientific, Click-iT® Cell Reaction Buffer Kit, Catalog no. C10269) applying CuAAC^27^. In brief, cells were washed with 1–3% bovine serum albumin (BSA) and incubated with the azide chromophore Alexa 488 for click chemistry reactions (Thermo Fisher Scientific) (Fig. 3). The cell reaction buffer contained Tris-buffered saline and 1 mM copper (II) sulfate (CuSO_4_), as well as an additive according to the manufacturer’s instructions (60 min at room temperature in the dark). Cells were finally washed in 1–3% BSA. The centrifuged cells were air-dried on coverslips (0.17 ± 0.01 mm). The coverslips were subsequently mounted on slides using antifade solution (Prolong Antifade Mountant, Thermo Fisher Scientific), followed by drying for 48 h at room temperature in the dark before storage at 4 °C.

### Microscopic analysis

In general, cells were enumerated, and signals were quantified compared with cells treated under identical conditions but grown under unfed control conditions using standard epifluorescence microscopy (Olympus BX53): green wavelength for AF, 530–550 nm (Emission 575–625 nm) and blue wavelength Alexa Fluor 488: 460–495 nm (Emission 510–550 nm). Cells were counted using the exposure time automatically adjusted for AF and applying the same exposure time to Alexa Fluor 488 emission for visualizing the peptide signal. Cell numbers (filament length) were estimated using standard image analysis. With this technique, hundreds of cells/filaments were inspected as selected from random images following standard counting protocols (Fig. 5).

High-resolution microscopy was performed using a laser scanning microscopy DMI 6000 SP8 (Leica Microsystems) with a 100 × HCX Plan Apo 1.4 oil objective. In brief, cells were inspected using a low energy white light laser (WLL, 70% output) with 498 nm excitation (7% output, collecting the Alexa Fluor 488 emission from 504–580 nm) and 620 nm excitation (7% output, collecting the red AF emission from 680–730 nm). The emission was recorded by hybrid detectors with low gain, a pinhole of 0.7 and an XY resolution of 50 nm per pixel. The Z resolution was aimed at 130 nm per pixel. Images were processed using Huygens Professional version 17.10 resulting in deconvolution of the recorded signals and higher resolution (Scientific Volume Imaging (SVI), VB Hilversum, The Netherlands, http://svi.nl). Images were deconvolved using the Classic Maximum Likelihood Estimation (CMLE) algorithm, with signal-to-noise ratio (SNR) = 25 and SNR = 7 for Alexa Fluor and AF, respectively. Using the same software, total (maximum) signal intensities and colocalization coefficients for pixels from two channels were calculated^52^. The Pearson colocalization coefficient ranged from −1 to 1, indicating either a perfectly opposite distributions of pixels or a perfectly similar distribution.

### Flow cytometry analysis

Using aliquots of cell preparations used for microscopy, we applied flow cytometry to estimate the number of stained cells/filaments resulting from peptide labeling. Fluorescence data were recorded using the Attune Acoustic Focusing Cytometer (Life Technologies, Thermo Fisher Scientific) equipped with violet (VL, excitation at 405 nm, 50 mW), blue (BL, 488 nm, 50 mW), and yellow (YL, 561 nm, 50 mW) solid-state lasers. Forward scatter and BL1 fluorescence (bandpass filter at 530 ± 15 nm) were used as triggers for all measurements to discriminate stained and nonstained particles at a flow rate of 0.1 ml min^−1^. The acquisition volume was 0.5 ml. Target particles (unlabeled control cells and peptide labeled cells) were quantified by gating on side scatter.

### Visualization of cellular compartments using standard immunofluorescence techniques

Three primary antibodies (polyclonal, raised in rabbit) were used: RbcL (Rubisco large subunit, form I and form II) for cytoplasm in cyanobacteria (AS03037), PsbA (D1 protein of PSII, C-terminal) as a thylakoid membrane marker (AS05084), and FtsZ (Procaryotic cell division GTPase) again for the cytoplasm (AS07217), (Agrisera, Sweden). To facilitate antibody binding, cells were harvested as described above, fixed with 96% EtOH (−20 °C) for 15 min, washed in PBS and stored at 4 °C^53^. Cells were immobilized on cover slips by air-drying for a few minutes, blocked for 30 min in PBS (2% BSA at RT) and incubated for 60 min with primary antibody diluted 1:500 in PBS (2% BSA at RT). After washing in PBS (3×), cells were incubated for 60 min with secondary anti-rabbit antibody Alexa Fluor 488 (#A11008, Thermo Fisher Scientific) diluted 1:250 in PBS (2% BSA at RT) in the dark. After washing in PBS (3×) and in Millipore water, cells were air-dried for a few min and mounted on slides using antifade solution (see above), Supplemental Fig. 5. For double localization of both clicked peptides and antibodies, cells were fixed in 96% EtOH (−20 °C) for 15 min and subsequently used for the click chemistry reaction using CuAAC (as described above). In a second step, the same cells with labeled peptides were again immobilized on cover slips by air-drying and used for immunofluorescence staining following the above protocol but using Alexa Fluor 405 (A-31556, Thermo Fisher Scientific). Cells were inspected using a sequential scan under the WLL conditions for 495 nm and 620 nm excitation as described above and a 405 nm diode laser (Em 415–584 nm). Pilot tests revealed no emission of Alexa Fluor 488 using 405 nm excitation.

Images were deconvoluted using the same Classic Maximum Likelihood Estimation (CMLE) algorithm, with SNR 7, 25 and 7 for Alexa Fluor 405, 488, and AF, respectively, and analyzed using Pearson colocalization coefficients. All quantitative measurements were statistically compared using Kruskal-Wallis One Way Analysis of Variance on Ranks followed by pairwise post hoc comparison (Sigma Plot 14.0).

To test for the uncontrolled loss of peptides by the EtOH fixation procedure, the signal intensities of peptides vs AF were compared for individual cells (n = 20) for all treatments as well as IF staining and compared with the signal intensities obtained from PFA-fixed cells. Total signal intensities per cell were calculated using the Ortho Slicer tool in Huygens from the histogram showing the number of pixels in the complete image plotted against the intensity value (Supplemental Figs. 6 and 7**)**.

## Supplementary information

Supplemental Info.

## Data Availability

All image files analyzed during the current study have been submitted to BioStudies under the Accession number S-BSST332 (www.ebi.ac.uk/biostudies).
