# Supplementary material for: Chemically labeled toxins or bioactive peptides show a heterogeneous intracellular distribution and low spatial overlap with autofluorescence in bloom-forming cyanobacteria
Source: Sci Rep. 2020 Feb 17;10:2781. doi: 10.1038/s41598-020-59381-w (PMC7026079; doi:10.1038/s41598-020-59381-w)
Supplement: Supplementary file 1 — Supplemental Info. [file 41598_2020_59381_MOESM1_ESM.docx]

**Supplemental Information**

**Chemically labeled toxins or bioactive peptides show a heterogeneous intracellular distribution and low spatial overlap with autofluorescence in bloom-forming cyanobacteria**

by

Rainer Kurmayer^1*^, Elisabeth Entfellner^1^, Thomas Weisse^1^, Martin Offterdinger^2^, Andrea Rentmeister^3^, Li Deng^4^

^1^University of Innsbruck, Research Department for Limnology, Mondseestrasse 9, 5310 Mondsee, Austria

^2^Innsbruck Medical University, Division of Neurobiochemistry, Biooptics Core Facility, Innrain 80, 6020 Innsbruck, Austria

^3^University of Muenster, Department of Chemistry, Institute of Biochemistry, Wilhelm-Klemm-Strasse 2, 48149 Muenster, Germany

^4^Helmholtz Centre Munich, Institute of Virology, Ingolstaedter Landstrasse 1, 85764 Neuherberg, Germany

*Corresponding author:

Rainer Kurmayer

University of Innsbruck

Research Department for Limnology

Mondseestrasse 9

5310 Mondsee, Austria

Phone: 0043 512 507 50242

Email: [rainer.kurmayer@uibk.ac.at](mailto:rainer.kurmayer@uibk.ac.at)

**Table S1:** Comparison of protonated masses [M+H]^+^ recorded from LC-MS chromatograms for *Planktothrix agardhii* grown at concentration of 0.05 mM of Prop-Lys or Prop-Tyr or control (three biological replicates). [M+H] measurements recorded in all three replicates, in two replicates or only once are indicated in bold, italics or in normal font, respectively. New (modified) molecules are listed in red. Fractions numbered 1-20 as in **Figure 4A-B**.

|  |  | Control |  | Prop-Lys |  | Prop-Tyr |  |  |
| --- | --- | --- | --- | --- | --- | --- | --- | --- |
| Fractions | RT [min] | m/z | z | m/z | z | m/z | z | Peptide |
| 1 | 4.7 | **821.3** | 1+ | **821.3** | 1+ | **821.3** | 1+ | Aer-type |
|  |  | **741.4** | 1+ | **741.4** | 1+ | **741.4** | 1+ | M - SO_3_ |
| 2 | 5.3 | **845.3** | 1+ | **845.3** | 1+ | **845.3** | 1+ | Aer-type |
|  |  | **765.4** | 1+ | **765.4** | 1+ | **765.4** | 1+ | M - SO_3_ |
| 3 | 13.1 | **609.3** | 1+ | **609.3** | 1+ | **609.3** | 1+ | Aer-type |
| 4 | 15.4 | 680.4 | 2+ | 680.4 | 2+ | 680.4 | 2+ |  |
| 5 | 16.4 | **809.5** | 1+ | **809.5** | 1+ | **809.5** | 1+ | AP-C |
| 6 | 16.9 | **823.5** | 1+ | **823.5** | 1+ | *823.5* | 1+ | AP-type |
| 7 | 17.3 | **837.5** | 1+ | **837.5** | 1+ | **837.5** | 1+ | AP-B |
|  |  | **419.2** | 2+ | **419.2** | 2+ | **419.2** | 2+ | AP-B (2-fold charged) |
|  |  | **504.2** | 2+ | **504.2** | 2+ | **504.2** | 2+ |  |
| 8 | 19.0 | **637.4** | 1+ | **637.4** | 1+ | **637.4** | 1+ | AP fragment |
| 9 | 21.6 | **844.4** | 1+ | **844.4** | 1+ | **844.4** | 1+ | AP-A |
| 10 | 21.8 | 809.5 | 2+ | 809.5 | 2+ | 809.5 | 2+ |  |
| 11 | 22.2 | **812.4** | 1+ | **812.4** | 1+ | **812.4** | 1+ |  |
| 12 | 24.9 | **629.4** | 1+ | 629.4 | 1+ | 629.4 | 1+ |  |
|  |  | **599.3** | 1+ | *599.3* | 1+ | 599.3 | 1+ |  |
| 13 | 25.1 |  |  | **891.5** | 1+ |  |  | Prop-AP-C |
| 14 | 25.9 | **1111.4** | 1+ | **1111.4** | 1+ | **1111.4** | 1+ |  |
|  |  | **1009.5** | 1+ | **1009.5** | 1+ | **1009.5** | 1+ |  |
|  |  | **991.5** | 1+ | **991.5** | 1+ | **991.5** | 1+ |  |
|  |  | **496.2** | 2+ | **496.2** | 2+ | **496.2** | 2+ | (2-fold charged) |
| 15 | 26.6 | 599.3 | 1+ | 599.3 | 1+ | 599.4 | 1+ |  |
| 16 | 27.9 | **613.4** | 1+ | **613.3** | 1+ | **613.4** | 1+ |  |
| 17 | 28.6 | **579.4** | 1+ | **579.4** | 1+ | **579.4** | 1+ |  |
| 18 | 29.5 | *627.4* | 1+ | 627.4 | 1+ | *627.4* | 1+ |  |
| 19 | 31.6 | **647.3** | 1+ | **647.3** | 1+ | **647.3** | 1+ |  |
|  |  | **902.9** | 2+ | **902.9** | 2+ | **902.4** | 2+ | Microviridin W(2-fold charged) |
| 20 | 31.9 |  |  |  |  | **882.4** | 1+ | Prop-AP-B |

**Table S2**: Original and modified anabaenopeptin (AP 891+AP 882) peptide structural variants and their MS fragments detected in *Planktothri*x *agardhii* No371/1. Fragmentation according to Entfellner et al. (2017). Erhard et al. (1999). Harms et al. (2016) and Spoof et al. (2016).

| [M+H]^+^ | n(MS) | Sequence | AP C | AP B | AP A | AP 891 | AP 882 |
| --- | --- | --- | --- | --- | --- | --- | --- |
| [M+H]^+^ | 1 |  | 809.5 | 837.6 | 844.6 | 891.6 | 882.6 |
| [M+Na]^+^ | 1 |  | 831.5 | 859.6 | 866.6 | 913.6 |  |
| Ret.time (min) |  |  | 16.3 | 17.1 | 21.6 | 25 | 32 |
| 637.4 | 2 | Lys+Val+Hty+MAla+Phe+2H | y | y | y | y | y |
| 619.4 | 3 | Lys+Val+Hty-H_2_O+MAla+Phe+H | y | y | y | y | y |
| 534.4 | 3 | Phe+Lys+Val+Hty-H_2_O+H | y | y | y | y | y |
| 460.4 | 3 | MAla+Phe+Lys+Val+2H | y | y | y | y | y |
| 405.2 | 3 | Hty+Val+Lys +2H | y | y | y | y | y |
| 387.2 | 3 | Lys+Val+Hty-H_2_O+H | y | y | y | y | y |
| 370.2 | 3 | Lys+Val+Hty-H_2_O-NH_2_+H | y | y | y | y | y |
| 233.0 | 3 | MAla+Phe+H |  | y | y |  |  |
| 201.0 | 2 | CO+Arg |  | y |  |  |  |

y, present

**Table S3:** Comparison of protonated masses [M+H]^+^ recorded from LC-MS chromatograms for *Microcystis aeruginosa* grown at concentration of 0.05 mM of Prop-Lys or Prop-Tyr or control (three biological replicates). [M+H] measurements recorded in all three replicates, in two replicates or only once are indicated in bold, italics or in normal font, respectively. New (modified) molecules are listed in red. Fractions numbered 1-16 as in **Figure 4C-D**.

|  |  | Control |  | Prop-Lys |  | Prop-Tyr |  |  |
| --- | --- | --- | --- | --- | --- | --- | --- | --- |
| Fractions | RT [min] | m/z | z | m/z | z | m/z | z | Peptide |
| 1 | 6.3 | *635.4* | 1+ | *635.4* | 1+ | **635.4** | 1+ |  |
|  |  | **621.5** | 1+ | **621.5** | 1+ | **621.4** | 1+ | Aer-type |
|  |  | **603.5** | 1+ | **603.5** | 1+ | **603.4** | 1+ | M - H_2_O |
| 2 | 8.2 | **663.5** | 1+ | **663.5** | 1+ | **663.4** | 1+ | Aer-type |
|  |  | **645.5** | 1+ | **645.5** | 1+ | **645.4** | 1+ | M - H_2_O |
| 3 | 11.0 | **645.5** | 1+ | **645.5** | 1+ | **645.4** | 1+ | Aer-type |
| 4 | 12.4 | **645.5** | 1+ | **645.5** | 1+ | **645.4** | 1+ | Aer-type |
| 5 | 24.1 | **773.0** | 2+ | **772.9** | 2+ | **772.9** | 2+ |  |
| 6 | 28.4 | *961.6* | 1+ | *961.6* | 1+ | *961.4* | 1+ |  |
|  |  | **899.6** | 1+ | **899.5** | 1+ | **899.4** | 1+ |  |
|  |  | **819.6** | 1+ | **819.6** | 1+ | **819.5** | 1+ | M - SO_3_ |
|  |  | *859.7* | 1+ | *859.7* | 1+ | *859.4* | 1+ |  |
| 7 | 29.3 |  |  |  |  | **1015.4** | 1+ |  |
|  |  |  |  |  |  | **953.5** | 1+ |  |
|  |  |  |  |  |  | **873.5** | 1+ | M - SO_3_ |
| 8 | 29.6 | 271.2 | 1+ | *271.2* | 1+ | *271.2* | 1+ |  |
| 9 | 31.2 | **1031.8** | 1+ | **1031.7** | 1+ | **1031.6** | 1+ | Asp-MC-YR |
|  |  | *516.3* | 2+ | **516.4** | 2+ | *516.3* | 2+ | 2-fold charged |
|  |  |  |  |  |  | 873.5 | 1+ |  |
| 10 | 32.8 | **1045.8** | 1+ | **1045.8** | 1+ | **1045.6** | 1+ | MC-YR |
|  |  | **523.4** | 2+ | **523.4** | 2+ | **523.3** | 2+ | 2-fold charged |
| 11 | 34.2 | **981.8** | 1+ | **981.8** | 1+ | **981.6** | 1+ | Asp-MC-LR |
|  |  | *491.3* | 2+ | *491.3* | 2+ | *491.3* | 2+ | 2-fold charged |
| 12 | 34.9 | **995.8** | 1+ | **995.8** | 1+ | **995.6** | 1+ | MC-LR |
|  |  | **498.4** | 2+ | *498.4* | 2+ | *498.3* | 2+ | 2-fold charged |
| 13 | 34.3 |  |  | *1078.5* | 1+ |  |  | Prop-Asp-MC-LR |
| 14 | 35.6 |  |  | *1092.6* | 1+ |  |  | Prop-MC-LR |
|  |  |  |  | 546.8 | 2+ |  |  | 2-fold charged |
| 15 | 39.4 |  |  |  |  | **1069.6** | 1+ | Prop-Asp-MC-YR |
|  |  |  |  |  |  | **535.3** | 2+ | 2-fold charged |
| 16 | 40.3 |  |  |  |  | **1083.6** | 1+ | Prop-MC-YR |
|  |  |  |  |  |  | **542.3** | 2+ | 2-fold charged |

**Table S4**: Original and modified microcystins (MC 1092. MC 1069 + MC 1083) peptide structural variants and their MS fragments detected in *Microcystis* *aeruginosa* strain Hofbauer. Fragmentation according to Fastner et al. 1999 and Welker et al. 2002.

| [M+H]^+^ | n(MS) | Sequence | Asp-YR | YR | Asp-LR | LR | 1069 | 1083 | 1092 |
| --- | --- | --- | --- | --- | --- | --- | --- | --- | --- |
| [M+H]^+^ | 1 |  | 1031.6 | 1045.6 | 981.6 | 995.6 | 1069.6 | 1083.6 | 1092.6 |
| [M+Na]^+^ | 1 |  | 1053.5 |  |  |  |  |  |  |
| Ret.time (min) |  |  | 30.9 | 32.4 | 33.8 | 34.2 | 38.9 | 39.8 | 35.6 |
| 958.6 | 1 | M-134 (Adda side chain) |  |  |  |  |  |  | y |
| 949.6 | 1 | M-134 (Adda side chain) |  |  |  |  |  | y |  |
| 935.5 | 1 | M-134 (Adda side chain) |  |  |  |  | y |  |  |
| 911.6 | 1 | M-134 (Adda side chain) |  | y |  |  |  |  |  |
| 897.5 | 1 | M-134 (Adda side chain) | y |  |  |  |  |  |  |
| 861.5 | 1 | M-134 (Adda side chain) |  |  |  | y |  |  |  |
| 847.5 | 1 | M-134 (Adda side chain) |  |  | y |  |  |  |  |
| 599.4 | 2 | Arg+Adda+Glu+H | y | y | y | y | y |  | y |
| 374.2 | 3 | C_11_H_14_O+Glu+Mdha+H | y | y | y |  |  |  |  |
| 238.5 | 3 | CO+Glu+Mdha-H |  | y |  |  |  |  |  |
| 213.0 | 3 | Glu+Mdha+H | y | y | y |  |  |  | y |
| 155.0 | 3 | Mdha+Ala+H | y | y | y |  |  | y | y |
| 136.1 | 3 | Tyr-CO+H | y | y |  |  |  |  |  |
| 135.0 | 1 | PhCH_2_CH(OCH_3_)-Adda side chain | y | y | y | y | y | y | y |

y, present


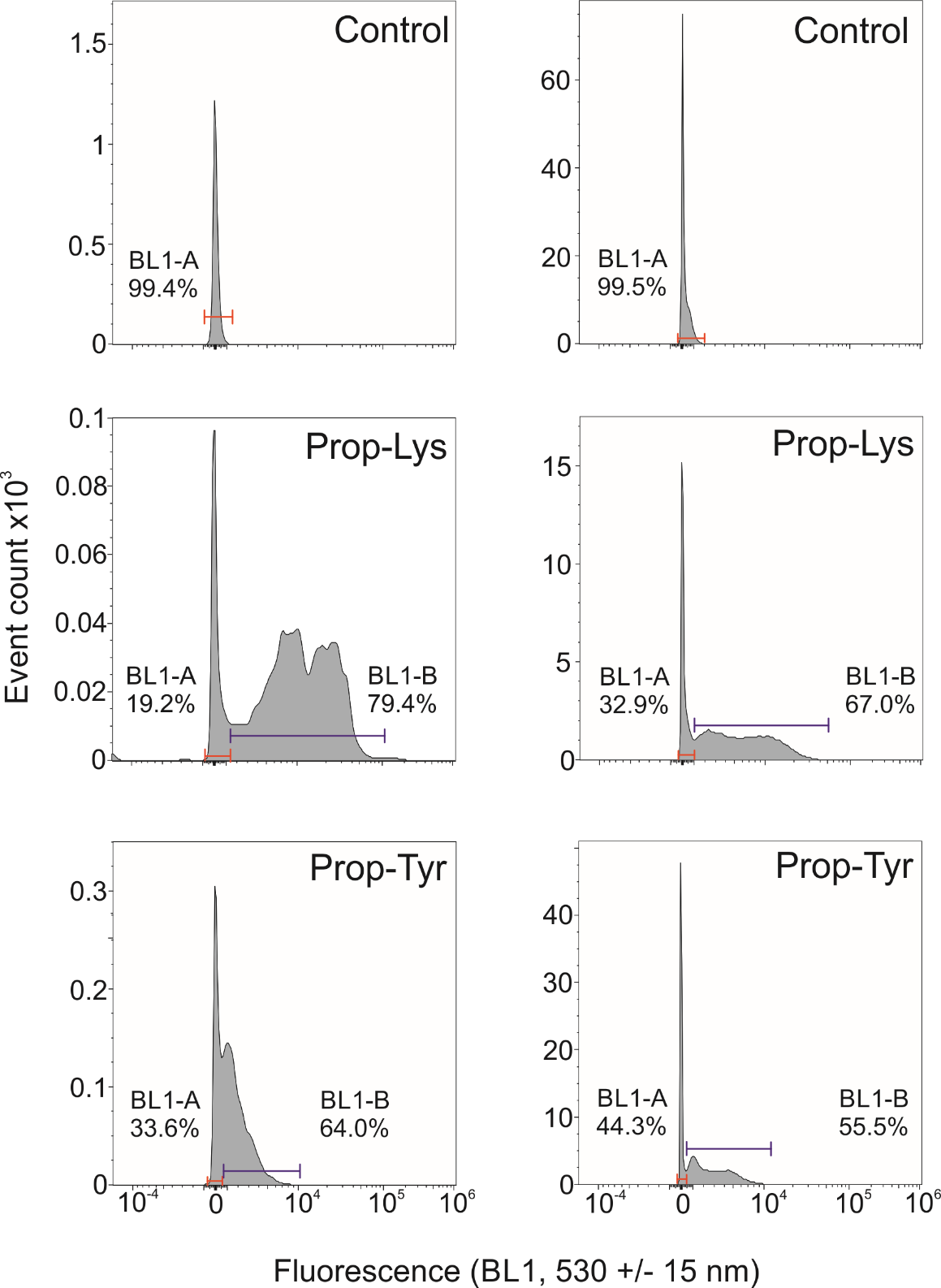


**Supplemental Figure 1.** Flow cytometry based detection of *Planktothrix agardhii* No371/1 filaments carrying labeled AP peptides resulting from Prop-Lys or Prop-Tyr using Alexa Fluor 488 alkyne as compared with control. Controls represent cells grown and processed for click chemistry under identical conditions but without substrate. Different colors through the indicated gates mark unlabeled cells (as inferred from controls) or labeled cells (BL1-A; BL1-B) and the respective proportion is indicated.


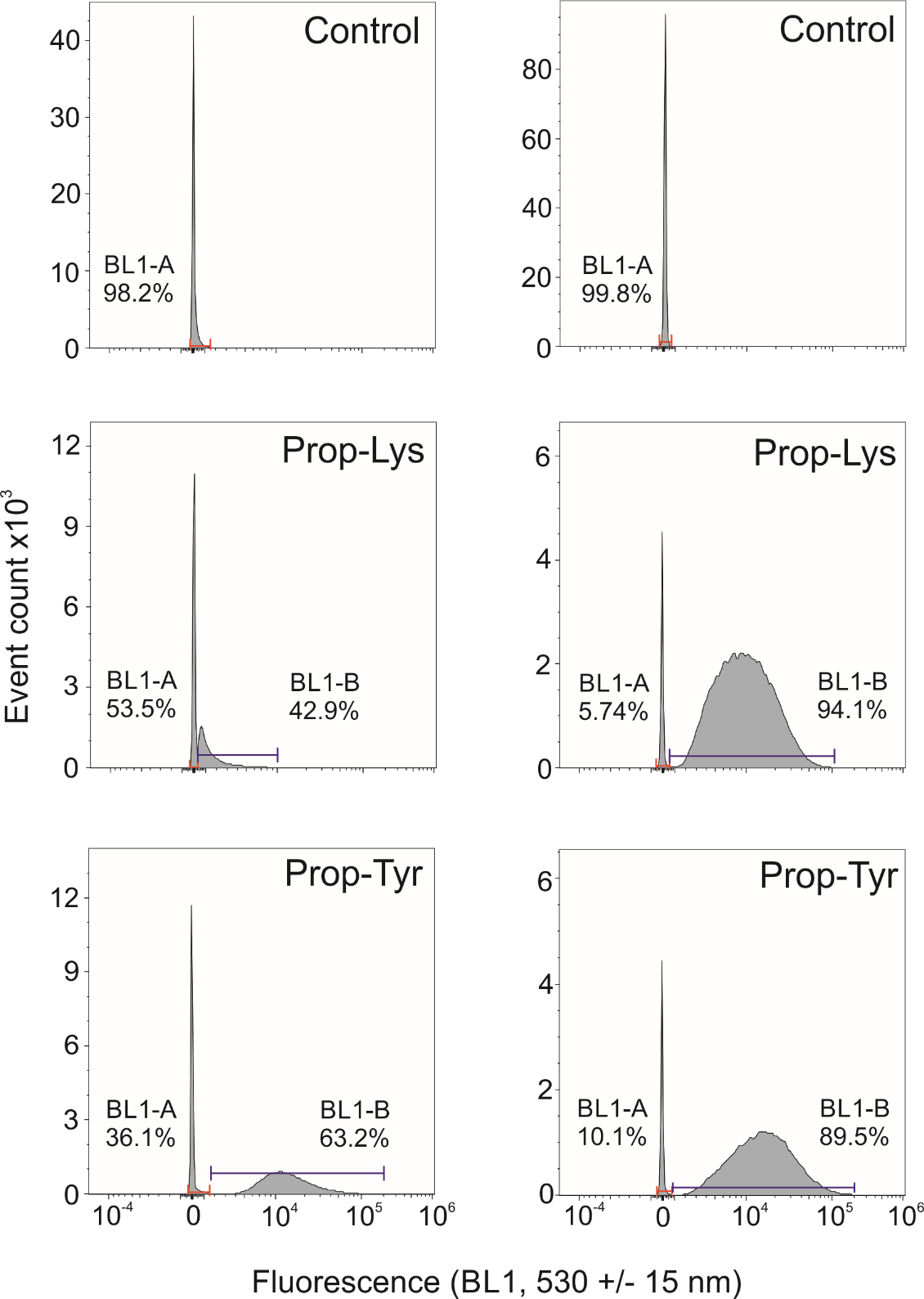


**Supplemental Figure 2.** Flow cytometry based detection of *Microcystis aeruginosa* cells carrying labeled AP peptides resulting from Prop-Lys or Prop-Tyr stained using Alexa Fluor 488 alkyne as compared with control. Controls represent cells grown and processed for click chemistry under identical conditions but without substrate. Different colors through the indicated gates mark unlabeled cells (as inferred from controls) or labeled cells (BL1-A; BL1-B) and the respective proportion is indicated. **
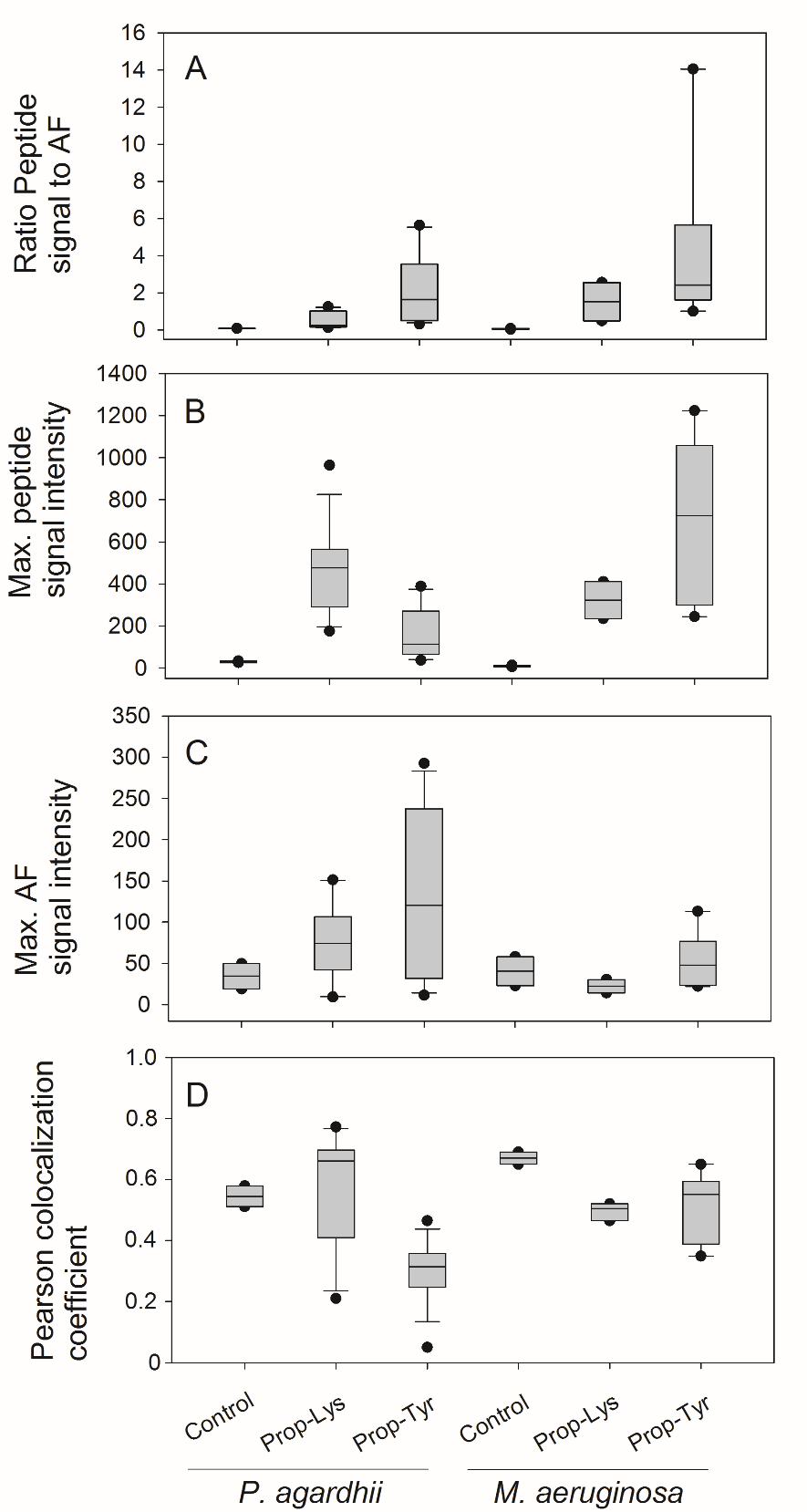
**

**Supplemental Figure 3.** (A) Ratio of the green peptide signal vs autofluorescence (AF) signal for *P. agardhii* No371/1 filaments and *M. aeruginosa* cells carrying labeled peptides resulting from Prop-Lys or Prop-Tyr using Alexa Fluor 488 alkyne as compared with control. Analogously maximum signal intensities for (B) AP or MC peptide signal and (C) AF are shown. (D) Pearson colocalization correlation coefficients between both signals indicating a decreasing match in distribution. Signal intensities and colocalization were calculated using the Huygens software (Scientific Volume Imaging (SVI), VB Hilversum, The Netherlands, http://svi.nl).


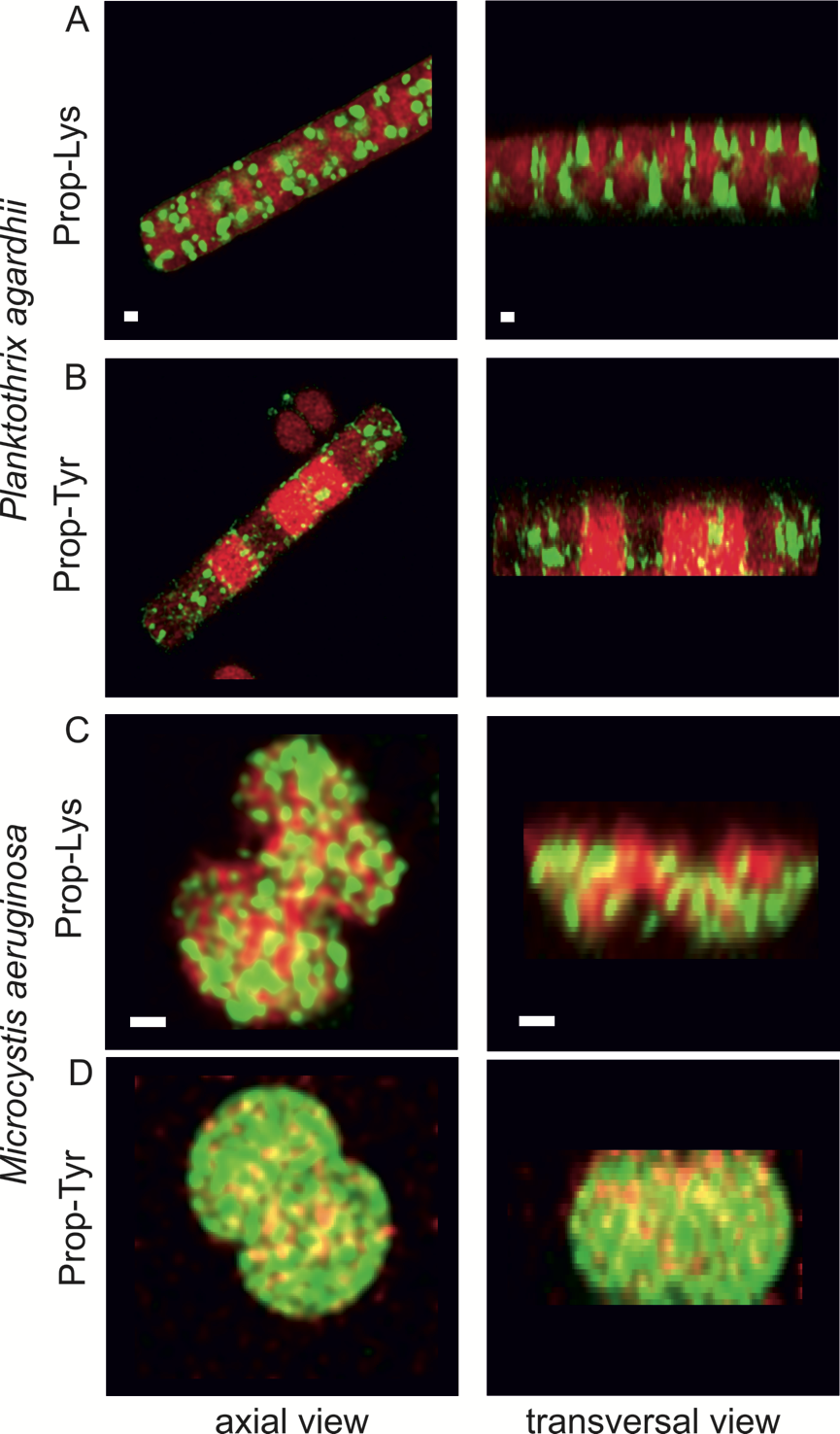


**Supplemental Figure 4**. Axial (left) and transversal (right) view of *Planktothrix agardhii* No371/1 cells synthesizing (A) new AP-Lys-alkyne [M+H]^+^ 891.6 and (B) AP-Tyr-alkyne [M+H]^+^ 882 and *M. aeruginosa* cells strain Hofbauer synthesizing (C) new MC-Lys-alkyne [M+H]^+^ 1092.6 or (D) DM-MC-Prop-Tyr-alkyne [M+H]^+^ 1069.5 and MC-Prop-Tyr-alkyne [M+H]^+^ 1083.5. Laser scanning microscope settings as described in **Figure 7**. The scale bar denotes one µm.

**
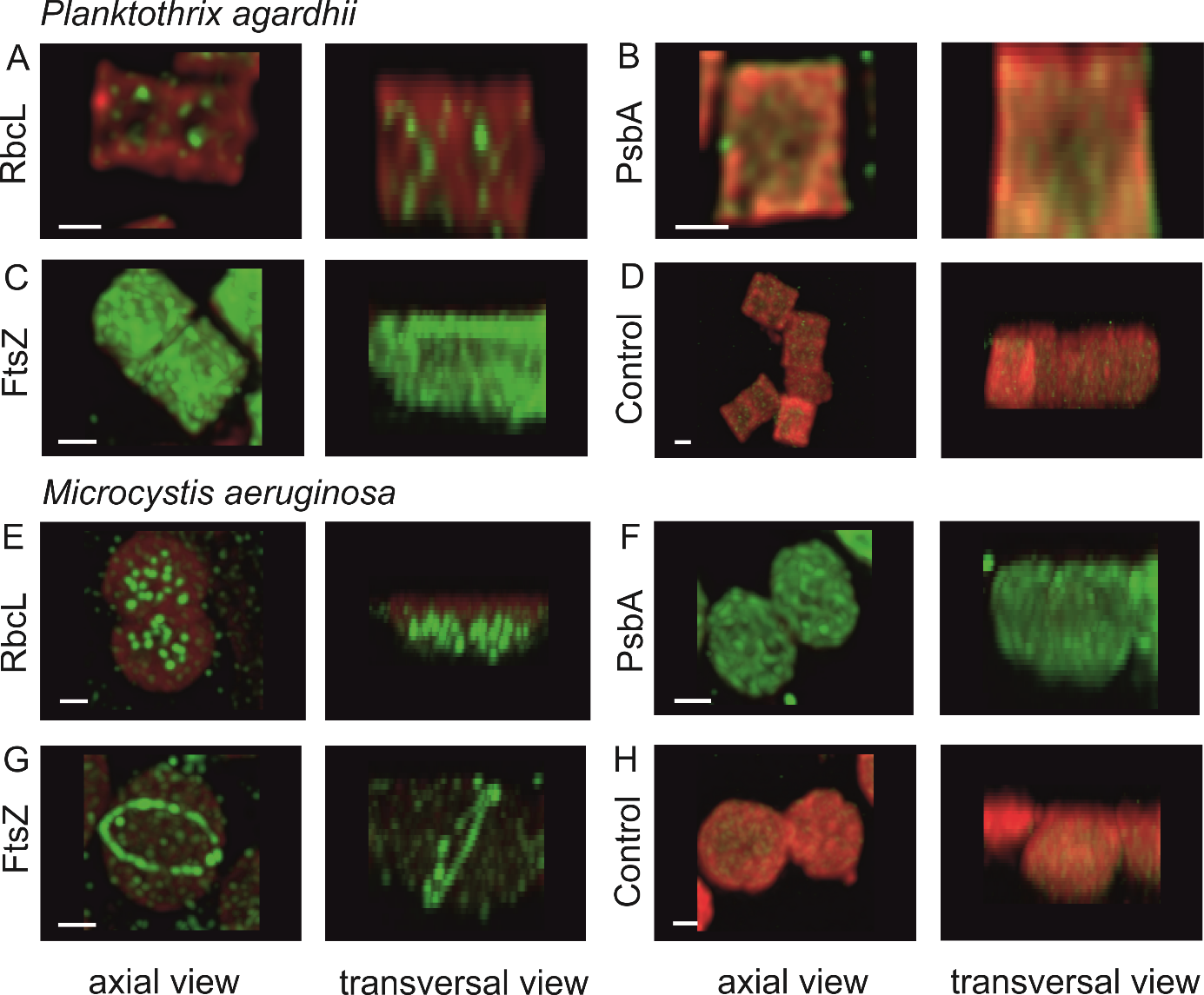
**

**Supplemental Figure 5.** Visualization of cellular compartments using the Alexa Fluor 488 immunofluorescence technique (A-D) in *P. agardhii* and (E-H) in *M. aeruginosa*. RbcL, Rubisco (Ribulose-1,5-bisphosphate carboxylase/oxygenase), PsbA, D1 protein of PSII, FtsZ, Procaryotic cell division GTPase, controls (without primary antibody). green emission = immunofluorescence by Alexa 488 fluorophore; red emission = autofluorescence by chlorophyll and phycobilins (cells fixed in 96% EtOH). Leica SP8 laser scanning microscope settings as described in **Figure 7**. The scale bar denotes one µm.

**
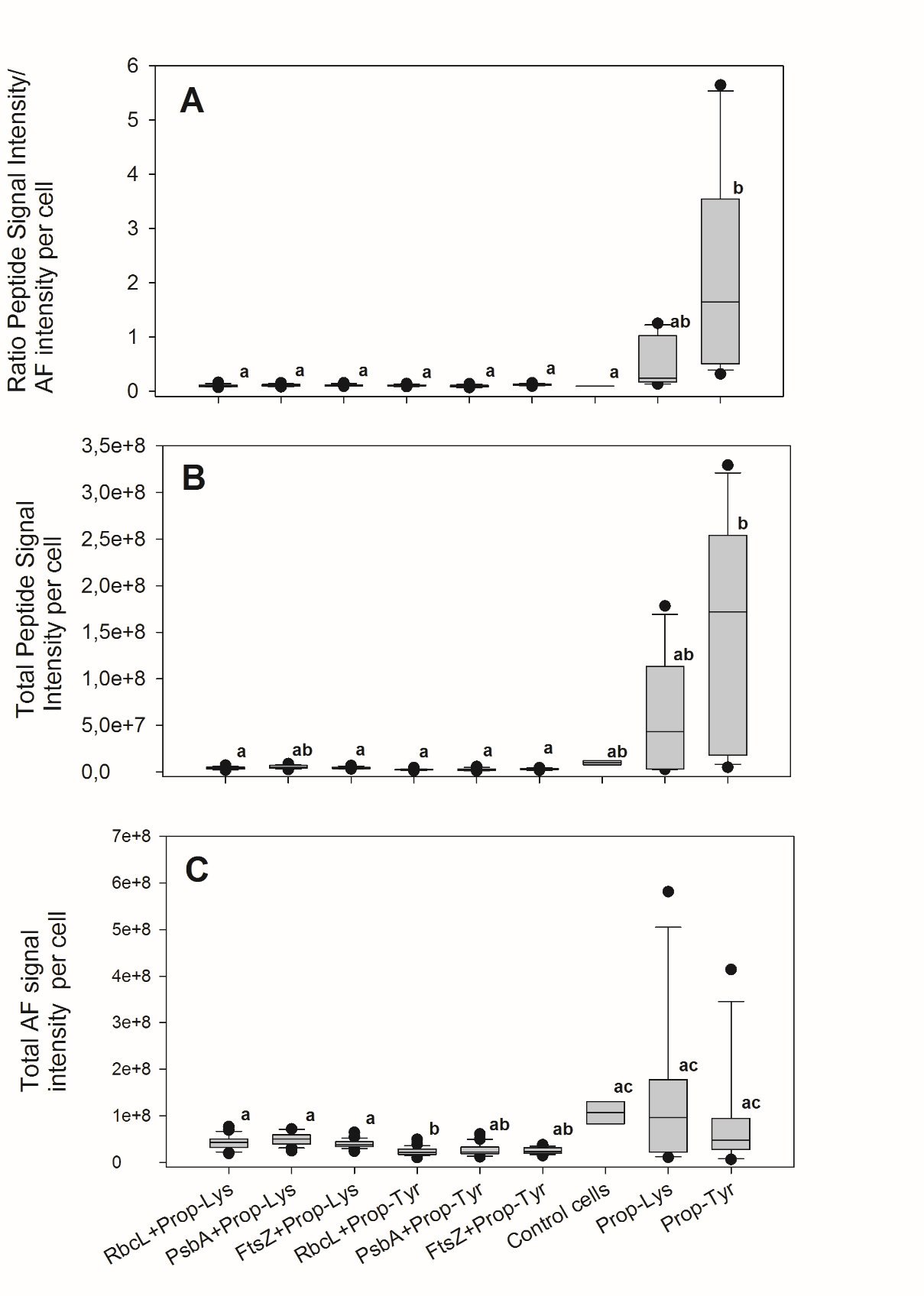
**

**Supplemental Figure 6.** *Planktothrix agardhii* 371/1 A) Ratio of total peptide vs AF signal intensity per cell, B) total peptide signal intensity per cell, C) total AF signal intensity per cell for cells fixed in EtOH and used for click-labelling and subsequent immunostaining as compared with cells fixed in PFA. Signal intensities were calculated using the Ortho Slicer tool in Huygens. Letters indicate subgroups not significantly different at p <0.05 if overall difference was found (n = 20).

**
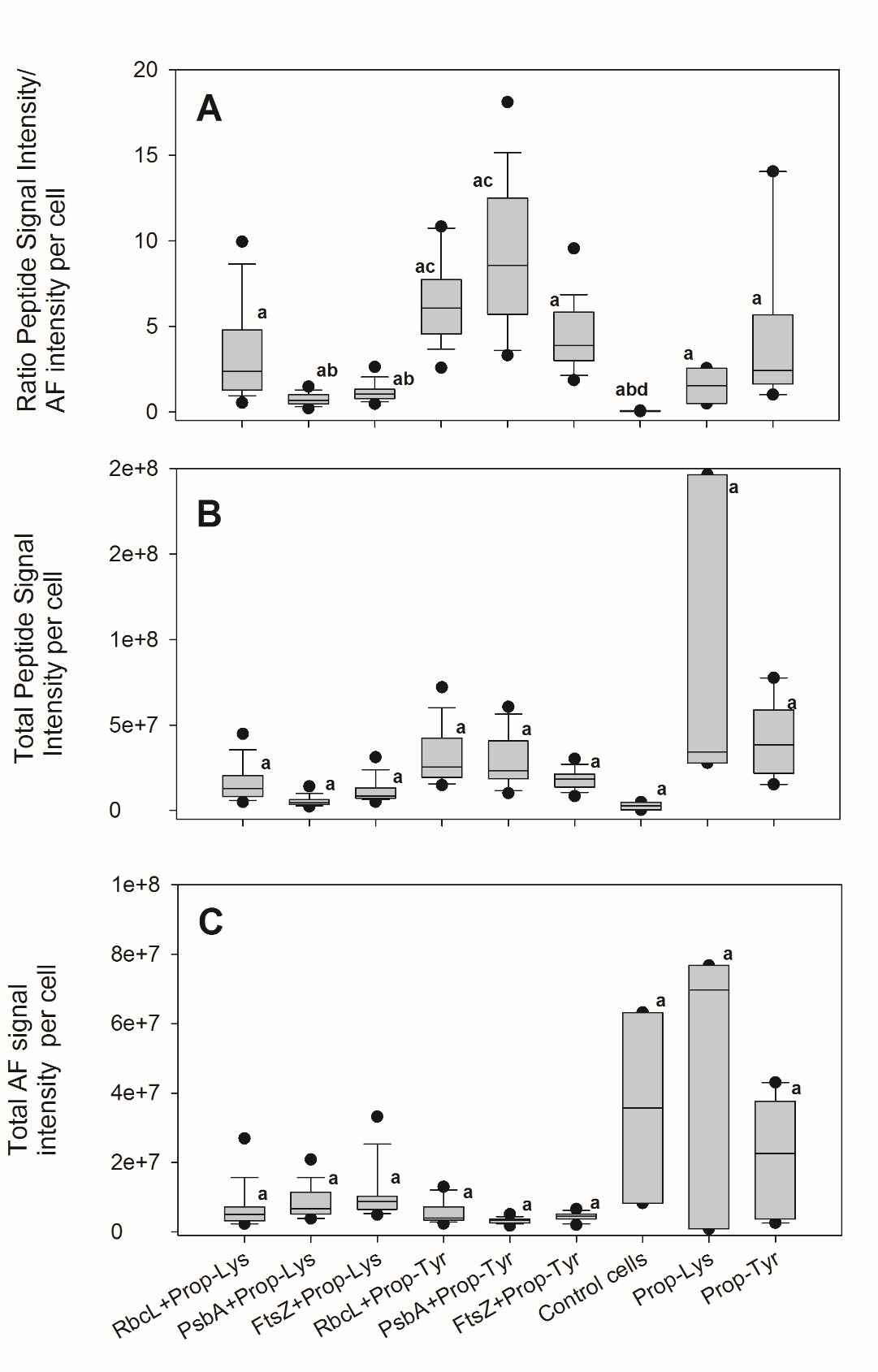
**

**Supplemental Figure 7.** *Microcystis aeruginosa* A) Ratio of total peptide vs AF signal intensity per cell, B) total peptide signal intensity per cell, C) total AF signal intensity per cell for cells fixed in EtOH and used for click-labelling and subsequent immunostaining as compared with cells fixed in PFA. Signal intensities were calculated using the Ortho Slicer tool in Huygens. Letters indicate subgroups not significantly different at p <0.05 if overall difference was found (n = 20).

**References Supplemental Information**

Entfellner. E.. M. Frei. G. Christiansen. L. Deng. J. Blom & R. Kurmayer. 2017. Evolution of anabaenopeptin peptide structural variability in the cyanobacterium *Planktothrix*. Frontiers in Microbiology 8(219) doi:10.3389/fmicb.2017.00219.

Erhard. M.. H. vonDöhren & P. Jungblut. 1999. Rapid identification of the new anabaenopeptin G from *Planktothrix* agardhii HUB 011 using matrix-assisted laser desorption/ionization time-of-flight mass spectrometry. Rapid Commun Mass Spectrom 13:337-343.

Fastner. J.. M. Erhard. W. W. Carmichael. F. Sun. K. L. Rinehart. H. Rönicke & I. Chorus. 1999. Characterization and diversity of microcystins in natural blooms and strains of the genera *Microcystis* and *Planktothrix* from German freshwaters. Arch Hydrobiol 145(2):147-163.

Harms. H.. K. L. Kurita. L. Pan. P. G. Wahome. H. Y. He. A. D. Kinghorn. G. T. Carter & R. G. Linington. 2016. Discovery of anabaenopeptin 679 from freshwater algal bloom material: Insights into the structure-activity relationship of anabaenopeptin protease inhibitors. Bioorganic & Medicinal Chemistry Letters 26(20):4960-4965 doi:10.1016/j.bmcl.2016.09.008.

Spoof. L.. A. Błaszczyk. J. Meriluoto. M. Cegłowska & H. Mazur-Marzec. 2016. Structures and Activity of New Anabaenopeptins Produced by Baltic Sea Cyanobacteria. Marine Drugs 14(1):8.

Welker. M.. J. Fastner. M. Erhard & H. von Döhren. 2002. Applications of MALDI-TOF MS analysis in cyanotoxin research. Environ Toxicol 17:367-374.
